# Cancer‐Associated Fibroblasts: Origin, Classification, Tumorigenicity, and Targeting for Cancer Therapy

**DOI:** 10.1002/mco2.70415

**Published:** 2025-10-28

**Authors:** Chang Fan, Wenlong Zhu, Yuan Chen, Wanwan Zhu, Jin Ding

**Affiliations:** ^1^ Clinical Cancer Institute Center For Translational Medicine Naval Medical University Shanghai China; ^2^ School of Gongli Hospital Medical Technology University of Shanghai for Science and Technology Shanghai China

**Keywords:** cancer‐associated fibroblasts, classification, drug resistance, therapeutic target, tumor microenvironment

## Abstract

Cancer‐associated fibroblasts (CAFs) are pivotal stromal components that shape the tumor microenvironment through their remarkable heterogeneity and dynamic functions. This review comprehensively examines the biology of CAFs, starting with their diverse cellular origins and induction mechanisms via key signaling pathways. We highlight the spatial‐temporal heterogeneity of CAF subsets and propose an updated classification system that encompasses eight functionally distinct subtypes based on recent single‐cell transcriptomic studies. The tumor‐promoting roles of CAFs are explored in depth, including their contributions to tumor behavior through novel mechanisms such as neutrophil extracellular traps formation, and multidrug resistance via metabolic reprogramming. A major focus is placed on CAF‐mediated immunotherapy resistance through immune checkpoint regulation, recruitment of immunosuppressive cells, and metabolite secretion. This review also highlights key technologies that are advancing CAF research, including patient‐derived 3D models, artificial intelligence‐enhanced spatial multiomics, clustered regularly interspaced short palindromic repeats (CRISPR)‐engineered mouse systems, and machine learning approaches for analyzing CAF heterogeneity and function. Finally, we evaluate therapeutic strategies targeting CAF activation, secreted factors, and immune crosstalk, while critically analyzing ongoing clinical trials. Overall, this review not only clarifies the biology of CAFs but also provides a translational framework for developing next‐generation stromal‐targeted therapies to overcome treatment resistance in cancer.

## Graphical abstract

This review explores the multifaceted roles of cancer‐associated fibroblasts (CAFs) in tumor biology. It examines their diverse origins, dynamic activation via signaling pathways, and spatial–temporal heterogeneity, proposing an updated classification of eight distinct CAF subtypes. The review highlights CAFs' contributions to tumor growth, metastasis, drug resistance, and immunotherapy resistance through mechanisms like NETs formation and immune checkpoint regulation. It also discusses advanced technologies like 3D organoids and AI‐driven spatial omics, and evaluates therapeutic strategies targeting CAFs for cancer treatment.

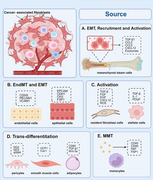

## 1 Introduction

Tumor initiation and progression trigger extensive, dynamic alterations in host tissues that ultimately yields a complex tumor stroma, commonly referred to as the tumor microenvironment (TME). In general, TME comprises tumor cells, infiltrating immune cells, fibroblasts, endothelial cells, adipocytes, extracellular matrix (ECM), and various cytokines and chemokines [1]. With advancing research on solid tumors, fibroblast populations within TME, collectively termed as cancer‐associated fibroblasts (CAFs), have been widely studied [2].Fibroblasts were originally described as spindle‐shaped cells that reside in connective tissues and synthesize collagens [3]. Given the absence of exclusive marker, CAFs are usually defined by α‐smooth muscle actin (α‐SMA) and fibroblast activation protein (FAP), the most commonly used markers in combination with cell morphology and tissue location in the existed studies [4]. Fibroblasts are the most resilient and versatile cells, sustaining tissue architecture and simultaneously participating in the wound healing processes of most organs. Quiescent fibroblasts are activated upon tissue injury [5]. Accompanied by the inflammation and fibrosis in tumors, the activated fibroblasts are converted into CAFs [6]. CAFs, the predominant stromal cells in the TME, not only interact with tumor cells to promote tumor progression but also influence other TME components by releasing cytokines and extracellular vesicles (EVs) [4]. CAFs exhibit substantial heterogeneity, originating from diverse cell types and characterized by the expression of factors such as α‐SMA, FAP, and fibroblast‐specific protein 1 (FSP1) [7]. Advances in single‐cell RNA sequencing (scRNA‐seq) have identified distinct CAF subgroups with unique functions and markers across solid tumors [1]. CAFs, activated by tumor cells, promote tumor growth, progression, angiogenesis, and drug resistance by secreting cytokines like transforming growth factor‐β (TGF‐β), platelet‐derived growth factor (PDGF), vascular endothelial growth factor (VEGF), and interleukins (ILs) [8]. The defining hallmark of CAFs is their unparallelled ability to synthesize the ECM and reshape TME. CAFs synthesize and secrete lots of type I, III, IV, and V collagen, fibrinolytic protein, hyaluronic acid (HA), and laminin [9], thus remodeling the local TME by promoting tissue hardening and stromal cell fibrosis [10]. The resulting dense scaffold acts as a physical barrier to immune infiltration and therapeutic penetration, yet simultaneously provides a suitable environment for the interaction between tumor cells and cytokines that accelerate malignant cell migration and invasion [11].

Recent studies highlight CAFs as key immune regulators in the TME. CAFs not only interact with tumor cells but also modulate immune cells within this niche. Multiple studies show that distinct CAF subpopulations regulate immune cells via different signaling pathways and reveal diverse mechanisms by which these subpopulations contribute to drug resistance during immunotherapy [8, 12, 13]. Additionally, CAFs serve as key regulators of critical metabolic processes in tumors, meeting the metabolic demands of rapidly proliferating tumor cells [14]. Researches into the TME have unveiled various mechanisms, presenting several potential targets for cancer therapy. However, clinical trials aimed at targeting CAFs have largely failed, and in some cases, have even exacerbated tumor progression [3]. This is largely because distinct CAF subpopulations exert divergent, and sometimes opposing, functions across different tumor types. Thus, understanding CAF subtypes and their roles in tumor immunity is crucial for developing CAF‐based targeted therapeutic strategies.

While the field has witnessed several comprehensive reviews addressing CAF biology and immunotherapy, important knowledge gaps remain. Rui et al. [15] provided a broad overview of cancer immunotherapies and their limitations, yet their discussion of stromal contributions was limited. Tsoumakidou [16] offered valuable insights into immunostimulatory CAF subsets but did not systematically examine resistance mechanisms. Kakarla et al. [17] highlighted CAFs as therapeutic targets but lacked the molecular resolution afforded by recent technological advances. This review systematically summarizes the different CAF subtypes based on the latest research, classifies and renames them, and discusses their heterogeneity and functions in promoting tumor progression, including proliferation, metastasis, and drug resistance. Importantly, the review elucidates the mechanisms by which CAFs participate in immune regulation and contribute to resistance in cancer immunotherapy. It also examines cutting‐edge technologies advancing CAF research and therapeutic strategies targeting CAF, while evaluating ongoing clinical trials. Collectively, this review provides an integrated framework that bridges fundamental CAF biology with clinical translation, offering both conceptual advances and practical strategies to overcome CAF‐mediated therapy resistance.

## 2 Origins of CAFs

The TME harbors a complex ecosystem where CAFs emerge as key stromal components through diverse cellular origins and differentiation pathways. Their formation represents a dynamic interplay between resident cell activation and recruited cell trans‐differentiation, creating substantial heterogeneity that complicates both biological understanding and therapeutic targeting.

### 2.1 Cellular Sources of CAFs

**FIGURE 1:**
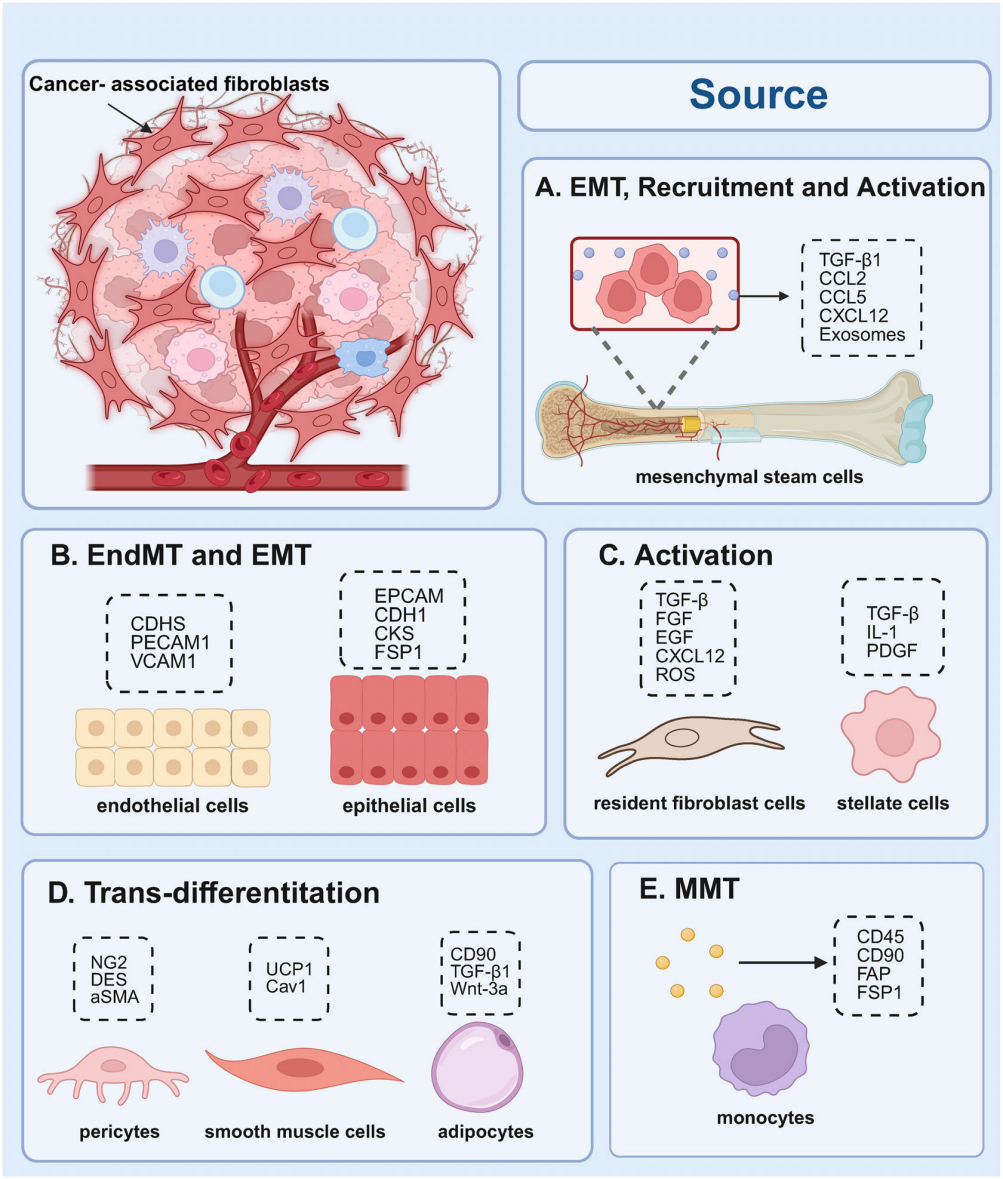
Cell of origin of cancer‐associated fibroblasts (CAFs). Schematic illustration of the potential cells of origin of CAFs that have been reported, including mesenchymal stem cells, endothelial cells, epithelial cells, resident fibroblasts, pericytes, smooth muscle cells, adipocytes, and monocytes.

The origins of CAFs remain poorly defined due to the absence of specific biomarkers. Lineage tracing studies suggest that CAFs can emerge from a variety of cellular precursors, with resident fibroblasts being the most common source [18]. These fibroblasts can be activated by internal signaling and external pressures, transitioning from a quiescent to a proliferative state. This transformation is driven by various cytokines in the TME, such as TGF‐β, FGF, EGF, and inflammatory chemokines‐along with hypoxic conditions [19, 20, 21]. For example, in breast cancer, TGF‐β activates the TGF‐β/Smad signaling pathway, promoting myofibroblastic CAFs (myCAFs) formation and stimulating CXCL12 secretion, which creates a positive feedback loop through CXCR4 activation [22]. Osteopontin produced by breast cancer cells can bind to CD44 and αvβ3 integrin on fibroblasts, activating the Akt and ERK pathways and inducing Twist 1‐dependent genes upregulation, leading to myCAF formation [23]. Similarly, quiescent pancreatic stellate cells (PSCs) and hepatic stellate cells (HSCs) can develop into myCAF‐like phenotypes in response to TGF‐β, IL‐1, and PDGF [24, 25]. The stiffness of the ECM, reactive oxygen species, DNA damage, and ultraviolet irradiation can also induce the formation of tumor‐promoting fibroblasts [26, 27]. Epithelial and endothelial cells can acquire a fibroblastic phenotype through epithelial to mesenchymal transition (EMT) and endothelial‐to‐mesenchymal transition (EndMT), respectively, and are considered sources of CAFs [28]. Additionally, CAFs may originate from bone marrow‐derived mesenchymal stem cells (BM‐MSCs). Karnoub et al. [29] injected BM‐MSCs into mice with breast cancer and observed that these cells could be converted into CAFs, facilitating breast cancer metastasis through the paracrine signaling of CCL5. Adipocytes can also be transformed into CAFs by tumor cell‐derived Wnt3a, confirming that adipocytes are another source of CAFs. Additionally, in non‐small cell lung cancer (NSCLC), CAFs can arise from monocytes via macrophage–myofibroblast transition, a process also implicated in fibrotic kidney disease [30]. Despite these insights, the exact origins of CAFs remain unclear due to shared lineages and a lack of specific markers. While genetic lineage tracing and fluorescence tagging have improved the study of cellular phenotype and type transformation, further research is needed to fully elucidate the biological origins of CAFs (Figure 1).

### 2.2 Heterogeneity of CAFs

Building upon their diverse cellular origins, CAFs exhibit extraordinary heterogeneity that manifests across multiple dimensions—from molecular signatures to functional specialization. In cancer, CAFs constitute a vital component of the tumor stroma, thereby drawing considerable attention to the feasibility of targeting CAFs as a therapeutic strategy for cancer treatment. However, CAFs, characterized by spatial, phenotypic, and functional heterogeneity, pose a significant challenge to targeted therapies. CAFs are predominantly characterized as spindle‐shaped cells that express α‐SMA, FAP, and FSP1, while conspicuously lacking epithelial, endothelial, or leukocyte markers [31]. Notably, these markers are nonspecific to CAFs and may vary among CAF subpopulations within the TME. Furthermore, the subtypes and functions of CAFs are markedly influenced by their tissue of origin—the type of cancer—since resident fibroblasts, the main precursors of CAFs, exhibit organ‐specific transcriptional profiles [18]. Given the presence of multiple local TMEs within a single tumor, activated CAFs can interact with tumor cells and other stromal cells across various interfaces, thereby modulating their plasticity in both spatial and temporal dimensions. In pancreatic ductal adenocarcinoma (PDAC), Öhlund et al. [32] identified two CAF subtypes with distinct transcriptional signatures: myCAFs and inflammatory CAFs (iCAFs). iCAFs show low α‐SMA expression and high expression of cytokines IL‐6, IL‐11, and PDGFRα, while myCAFs selectively express high levels of α‐SMA and lack inflammatory cytokines. Spatial distribution differences between these two CAF subtypes have been observed through immunostaining of tumor organoids, with myCAFs located near tumor cells and iCAFs located more distantly from the tumor edge. Subsequently, these subtypes were confirmed in a pancreatic cancer mouse model. They observed dynamic transitions between the subtypes, prompted by IL‐1 and TGF‐β signaling, thereby supporting the notion of distinct CAF subtypes within the same tumor [20]. Another study identified a novel CAF subtype in advanced pancreatic cancer, termed antigen‐presenting CAFs (apCAFs), which express MHC‐II‐related genes and CD74. Due to the lack of classical costimulatory molecules such as CD40, CD80, and CD86, apCAFs induce T cell anergy and promote the formation of regulatory T cells (Tregs) [33]. Consistent with these findings, apCAFs activate CD4^+^ T cells and drive their differentiation into Tregs, fostering an immunosuppressive tumor environment [34]. ScRNA‐seq of CAFs in different stages of KIC tumors revealed that mesothelial cells in normal pancreas share similar genetic features with apCAFs, suggesting a potential mesothelial origin for apCAFs [35]. Additionally, Wang et al. [36] uncovered a novel CAF subtype in loosely stromal PDAC tumors, characterized by a highly activated glycolytic metabolic state (termed meCAFs), which is associated with higher metastasis rates but paradoxically, a better response to immunotherapy.

Beyond PDAC, six CAF markers—FAP, CD29 (integrin‐β1), α‐SMA, FSP1, PDGFRβ, and caveolin—have delineated four subtypes (CAF‐S1 to CAF‐S4) in ovarian and breast cancers [37]. Utilizing scRNA‐seq, Kieffer et al. [38] further characterized eight CAF‐S1 subtypes in breast cancer (clusters 0–7). Among these, the ECM‐myCAF and TGF‐β‐myCAF subtypes play crucial roles in establishing an immunosuppressive microenvironment and facilitating immune evasion. Specifically, ECM‐myCAF boosts the number of Foxp3^+^ Treg cells and PD‐1 and CTLA‐4 expression on their surface, which in turn converts ECM‐myCAF to TGF‐β‐myCAF. Similarly, Bartoschek et al. [39] identified four CAF subtypes in the breast cancer TME: developmental CAFs (dCAFs) associated with tissue differentiation, matrix CAFs (mCAFs) linked to stromal genes, vascular CAFs (vCAFs) related to angiogenesis, and cycling CAFs (cCAFs) related to cell proliferation. These subtypes exhibit distinct cellular origins, with vCAFs, mCAFs, and dCAFs arising from perivascular regions, resident fibroblasts, and tumor cells undergoing EMT, respectively. The cCAFs primarily originate from the proliferation of vCAFs. These subtypes originate from different sources and have distinct functional profiles with clinical implications [39]. Additionally, breast cancer CAFs can be categorized into three classes: steady state‐like, mechanoresponsive, and immunomodulatory (IM) CAFs. Disrupting mechanotransduction or employing immunosuppressants alters the distribution of these subtypes, indicating a dynamic interplay among them [40]. Elucidating the spatial dynamics of CAF biology and the regulatory pathways of CAF differentiation could provide a valuable omics framework for future cancer research.

In NSCLC, Cords et al. [41] employed spatially resolved single‐cell imaging mass cytometry (IMC) to identify four CAF subtypes linked to clinical outcomes. Tumor‐like CAFs are associated with poor prognosis, while inflammatory and interferon‐response CAFs correlate with inflamed TMEs and enhanced survival rates. In contrast, matrix CAFs reduce immune infiltration and correlate with lower patient survival. This study provides a foundation for understanding how CAF phenotypes influence tumor growth and immune infiltration. In gastric cancer (GC), researchers identified a subtype of ECM CAFs (eCAFs), characterized by high expression of Periostin (POSTN), a protein involved in ECM remodeling and cell adhesion. And these eCAFs are strongly associated with poor outcomes in GC patients [42]. Meanwhile, Galbo et al. [8] identified six CAF subtypes common in melanoma, head and neck squamous cell carcinoma, and lung cancer. Their research implicates pan‐myCAF, pan‐dCAF, pan‐iCAF, pan‐pCAF, and pan‐iCAF‐2 subtypes in immunotherapy resistance.

The remarkable heterogeneity of CAFs, while reflecting their functional versatility, presents substantial obstacles for therapeutic development. Despite CAFs forming the majority of the tumor stroma, their clinical targeting has been impeded by the absence of specific markers and their pronounced heterogeneity. The lack of specific ligands for CAFs means that current therapies often fail due to nonspecific targeting of other cells. To overcome these challenges, researchers are employing single‐cell analysis, proteomics, genomics, and transcriptomics analysis to further characterize CAFs and identify markers of specific subpopulations, thereby enhancing cancer treatment efficacy.

## 3 New Classification and Function of CAFs in Tumor Immunity

The evolving understanding of CAF heterogeneity has revolutionized our perspective on their roles in shaping tumor immunity. Recent advances in single‐cell technologies have uncovered unprecedented diversity among CAF populations, revealing distinct subtypes with specialized IM functions that either support or counteract antitumor immunity. This section synthesizes cutting‐edge research to present an updated classification framework and examines how different CAF subtypes orchestrate immune responses within the TME.

### 3.1 New Classification of CAFs

CAFs, spindle‐shaped cells located within or adjacent to tumor tissues, exhibit significant heterogeneity due to their diverse origins. For instance, in PDAC, different normal fibroblast (NF) precursors give rise to various CAFs subtypes [43]. Elucidating the spectrum of CAF subtypes and their functional roles is crucial for addressing the challenge of immune resistance in cancer. However, there is no comprehensive classification to date given the emerging new subtypes of CAF. Traditionally, CAFs that express high levels of α‐SMA have been classified as myCAFs [44], whereas those that express high levels of CXCL12, IL‐6, and CXCL14, which possess IM functions, have been defined as iCAFs [35]. These two subtypes are not only spatially segregated but also capable of interconversion and mutual exclusion [45]. Elyada et al. [34] first defined CAFs with high expression of CD74 and MHC‐II as apCAFs. These three types of CAFs are currently the most predominant subtypes of CAFs. On this basis, Cords et al. [46] divided CAFs into five main subtypes. However, according to the latest research, this classification method can be further optimized. A comprehensive cross‐tissue human fibroblast atlas, integrating scRNA‐seq data from 517 samples, has revealed a more complex cellular and transcriptional landscape than previously known. Distinct myofibroblast subtypes, like LRRC15^+^ and MMP1^+^ cells, are linked to specific TMEs and have varied roles in immune modulation [13]. These findings highlight the complexity of CAF heterogeneity and the need for an updated classification system. This review synthesizes emerging evidence to propose a novel eight‐subtype classification that advances the field through several key improvements (Table 1). The new system comprehensively integrates both newly identified and established CAF populations, providing a more complete representation of tumor heterogeneity. Importantly, it establishes clear connections between each subtype and specific immune‐modulatory mechanisms, effectively bridging molecular characteristics with functional consequences. The classification's clinical relevance is particularly noteworthy, as it maps subtype‐specific markers to therapy resistance mechanisms, offering an actionable framework for developing CAF‐targeted strategies to restore antitumor immunity. Beyond resolving ambiguities present in earlier models, this refined classification introduces a dynamic understanding of CAF plasticity. By unifying molecular definitions with functional and therapeutic insights, the system represents a significant advancement in addressing CAF‐mediated immune resistance in cancer.

**TABLE 1:** New classification of CAFs.

CAFs	Tumor type	Subtype	Biomarkers	Function	Key pathway	References
MyCAF^a^	BRCA^h^		α‐SMA, FAP	Maintain tumor stable ECM remodeling	−	[47]
			PDPN, MME, TMEM158, NDRG1, VEGF‐a	Promote tumor proliferation	EMT‐related pathwayTGF‐β pathway KRAS pa thway	[41]
		ECM‐CAF	FN1, POSTN, CTSB, LOXL1, MMP14	ECM remodeling	−	[48]
	PAAD^i^		α‐SMA, FAP	Promote tumor proliferation	TGF‐β pathway	[32]
		LRRC15^+^ MyCAF	LRRC15	Immunosuppression	TGF‐β pathway	[49]
		MMP11^+^ CAF	MMP11	ECM remodeling	PI3K/AKT pathway	[50]
		COL17A1^+^ CAF	COL17A1	Promoted chemotherapy resistance	−	[51]
			COL1A1, COL1A2, FN1, POSTN	ECM remodeling	TGF‐β pathway	[52]
	COAD^j^		COL1A1, COL1A2, FAP, PDPN	Wound healing	−	[53]
		ECM MyCAF	GJB, ANTXR1, SDC1	Immunosuppression	−	[53]
		TGF‐β MyCAF	CST1, TGF‐β1, ANTXR1, LAMP5	Immunosuppression	−	[53]
		Wound MyCAF	SEMA3C, ANTXR1, CD9	Immune activation	−	[53]
		CD70^+^ CAF	α‐SMA, FAP	Promote migration	−	[54]
			α‐SMA, FAP, FSP‐1, vimentin	Promote EMT	Wnt/β pathway	[55]
		SFRP2^+^ CAF	SFRP2	ECM remodeling	−	[56]
		CTHRC1^+^ CAF	CTHRC1	Promote EMT	WNT5A–MSLN signaling pathway	[57]
	STAD^k^		α‐SMA, IGFBP‐7, FAP	Promote tumor invasion	−	[58]
		POSTN^+^ CAF	POSTN	Promotes cancer cell metastasis	−	[42]
			MMP 14, LOXL2, POX 2	Promote tumor invasion	−	[42]
		THBS2^+^ mCAF	THBS2, ACTA2, COL1A1, IGF1	Immunosuppression	C3–C3AR1 axis	[59]
	LUAD^l^		FAP, α‐SMA	Secreting collagen XI/XII repels T cells	−	[60]
		MYH11^+^ α‐SMA^+^ CAF	α‐SMA	Secreting collagen IV rejects T cells	−	[60]
		S1‐CAF	COL1A1, COL3A1, COL4A1, ACTA2, TPM2, MYH11, TAGLN, MYL9	ECM remodeling smooth muscle contraction	−	[61]
		CXCL14^+^ MyCAF	CXCL14	Promote EMT and angiogenesis	−	[62]
	LIHC^m^		ACTA2, COL1A1, COL1A2, COL1A3, COL15A1, MMP2	ECM remodeling	−	[63, 64]
		CAF‐FAP	FAP, POSTN, THBS2, NOX4, COL11A1	ECM remodeling	MAPK pathway	[65]
	BLCA^n^		RGS5, FAP, α‐SMA	−	−	[66, 67]
		COL18A1^+^ CAF	COL18A1	Promote recurrence	−	[68]
	CESC^o^		COL1A1, COL3A1, COL4A1, COL5A2, COL6A3	Blocking immune cell infiltration and crosstalk ECM remodeling	p53 pathway	[69]
	HNSC^p^		α‐SMA	Secrete growth factors and metabolizing factors	−	[70]
		PTN^+^ MyCAF	PTN	Promote CD8^+^ T cell infiltration	−	[71]
		COL5A2^+^ CAF	COL5A2	Enhance erlotinib resistance	PI3K/AKT pathway	[72]
	ESCA^q^		MMP 1, MMP 11, FAP, VIM, α‐SMA	Promote cancer cell progression	−	[73]
	RCC^r^		FAP, α‐SMA	ICIs primary resistance	−	[74]
	OC^s^		RGS5, NOTCH3, NDUFA4L2	−	−	[75]
			α‐SMA, vimentin, COL3A, COL10A, MMP11	Promote cancer cell invasion	−	[76]
	Melanoma		TAGLN, NDUFA4L2, RGS5, STEAP4, ACTA2	−	−	[77]
		CEMIP^+^ fibroblasts	CEMIP	ECM remodeling	−	[78]
		NKD1^+^ fibroblasts	NKD1	ECM remodeling	WNT pathway	[78]
	CHOL^t^	eCAF	KRT19, KRT8, SAA1	ECM remodeling	−	[64, 79]
		mCAF‐ECM	COL5A1, COL5A2, COL6A3	ECM remodeling	−	[64, 79]
		POSTN^+^ FAP^+^CAF	POSTN, FAP	ECM remodeling Promote tumor proliferation	−	[80]
	THCA^u^		α‐SMA, FAP, MMP	Promote cancer cell invasion and metastasis	−	[81]
	GBC^v^	TSP4^+^ CAF	α‐SMA	Promote cancer cell proliferation and EMT	PI3K/Akt pathway	[82]
	OSCC^w^		α‐SMA, FAP, vimentin	Promote cancer cell invasion, metastasis, and EMT	AKT/GSK3β/β‐catenin pathway	[83]
	PRAD^x^	CTHRC1^+^ CAF	CTHRC1	Promote TAM polarization	TGF‐β pathway	[84]
	Pan‐cancer	LRRC15^+^ CAF	LRRC15, FAP	Interact with M2‐polarized macrophages	−	[46]
ICAF^b^	BRCA^h^		PLA2G2A, CXCL12, CXCL14	Proinflammatory Immunomodulation	IL‐6–JAK–STAT3– KRAS pathway	[41]
		DKK1^+^ CAF	DKK1	Suppress NK cells	Downregulate AKT/ERK/S6 phosphorylation	[85]
		FAP^+^ CAF	FAP	Facilitate bone metastasis	MK pathway	[86]
	BRCA^h^ (mice)	pCAF	PDPN	Suppress NK cells	−	[87]
	PAAD^i^		IL‐6, CXCL1, CXCL2	Proinflammatory Suppressor T cells	IL‐6–JAK–STAT3 pathway	[32, 49]
	COAD^j^		CXCL12	Proinflammatory Immunomodulatory	−	[53]
		IL‐iCAF	SCARA5, DLK1	−	−	[53]
		Detox‐iCAF	ADH1B, GPC3	−	−	[53]
		IL‐1 R1^+^ CAF	IL‐1 R1	Immunosuppression	−	[88]
			CXCL12	Promote tumor invasion	PI3K/Akt pathway	[89]
	STAD^k^		CXCL12, IL‐6, CXCL14	Promote tumor invasion Immunosuppression	−	[42]
			α‐SMA, IL‐17a	Promote tumor invasion	JAK2/STAT3 pathway	[90]
		CCL2^+^ CAF	CCL2	Promote tumor invasion	JAK–STAT3 pathway	[91]
	LIHC^m^		IL‐6, HGF, BMP10, GDF2, LFITM1	Enhances tumor immune tolerance Stimulates monocytes to differentiate into MDSC	STAT3–PDL1 and IL‐6–STAT3 pathways	[92, 93] [63, 64]
			α‐SMA, IL‐6	Promote EMT	JAK/STAT3 pathway	[94]
		CAF‐C7	C7, PDGFRA	Complement activation	PI3K/Akt pathway	[65]
	GBC^v^		α‐SMA, FAP, IL‐6	Promote tumor proliferation	JAK/STAT3 pathway	[64]
	CHOL^t^		FBLN1, IGFI, CXCL1, C3, C7	Complement activation	−	[64, 79]
	BLCA^n^		PDGFR‐a, CXCL12, IL‐6, CXCL14, CXCL1, CXCL2	Promote tumor proliferation Immunosuppression	−	[66, 67]
		Interferon‐regulated CAFs	NRG1, STC1, WNT5A	Response interferon	−	[67, 95]
	HNSC^p^		IL‐6, CXCL12	Promotes cancer cell proliferation and metastasis Promotes angiogenesis	−	[70]
		CKS2^+^ iCAF	CKS2	Depleted CD8^+^ T cells	−	[71]
		IL‐11^+^ iCAF	IL‐11	Immunosuppression	NF‐kB pathway	[96]
	ESCA^q^		CXCL1, IL‐6, CXCL12, IL‐24, CXCL18	Promote tumor proliferation	−	[73, 97, 98]
		WNT2^+^ CAF	WNT2	Inhibition of CD8^+^ T cells Inhibits differentiation and maturation of DC	Suppress SOCS3/p‐JAK2/p‐STAT3 pathway	[99]
	Melanoma		FBLN1, COL5A1, LUM, DCN, COL6A3, DIO2, COL1A1	−	−	[77]
		PDPN^+^ CAF	PDPN	Promote tumor proliferation	−	[100]
	RCC^r^	CD248^+^ CAF	CD248	Immunosuppression	−	[101]
	THCA^u^		IL‐6, IL‐8, CXCL9, CXCL10	Immunosuppression	TGF‐β pathway	[81]
	LUAD^l^	HGF^+^ FGF7^+^ CAF	HGF, FGF7	Immunosuppression	−	[102]
		s2‐CAF	LIF, IL‐6	Immunosuppression	−	[61]
		FAP^+^ α‐SMA^+^ CAF	FAP, α‐SMA	Exhausted CD8^+^ T	−	[103]
		MYH11^+^ α‐SMA^+^ CAF	MYH11, α‐SMA	Immunosuppression Infiltrate CD4^+^ Treg	−	[103]
	Chordoma		CCL4, IL‐1B, MMP9	Immunosuppression	−	[104]
						
ApCAF^c^	BRCA^h^		MHC‐II, HLA‐DRA, CD74	Antigen presentation	−	[41]
	PAAD^i^		MHC‐II, CD74	Antigen presentation	−	[32]
	CHOL^t^		CD74, HLA‐DRA	Response interferon Antigen presentation	−	[64, 79]
	HNSC^p^		MHC‐II	Immunosuppression Antigen presentation	−	[70]
	ESCA^q^		MHC‐II, CD74	Promote proliferation of CD4^+^ Treg cells Activate CD4^+^ and CD8^+^ T cells	−	[99]
			C1QA, C1QB, MSR1, FCGR3A	Antigen presentation	−	[105]
	STAD^k^		MHC‐II	Strengthening T cell‐mediated antitumor immunity	−	[106]
	LUAD^l^		HLA‐DRA	Recruit FOXP1^+^ Treg	JAK1/2_STAT1–IFI6/27 pathway	[107]
VCAF^d^	BRCA^h^		NOTCH3, COL18A1, MCAM	Promotes angiogenesis	−	[41]
	CHOL^t^		CD146, MYH11	Promotes angiogenesis	−	[64, 79]
	CESC^o^		CD146, RGS5, MYH11	−	−	[108]
	COAD^j^	WNT2^+^ CAF	WNT2	Promote angiogenesis	−	[109]
	LIHC^m^	VEGFA^+^ CAF	VEGFA	Promote angiogenesis	−	[110]
	STAD^k^	MYH11^+^ CAF	MYH11	Promote angiogenesis	−	[111]
		PDPN^+^ CAF	PDPN	Promote angiogenesis	AKT/NF‐kB pathway	[112]
CCAF^e^	BRCA^h^		TUBA1B, MKI67	Promotes cell division	−	[41]
	BRCA^h^ (mice)		ki‐67	Promotes cell division	−	[39]
	STAD^k^	pCAF	TOP2A	Promote tumor proliferation	−	[106]
	ESCA^q^	pCAF	SNAP25, MAGEA3, CHGB	Promote tumor proliferation	−	[105]
	PRAD^x^	ABCA8^+^ CAF	GATA3, WT1, ZNF641, NFAT5, HOXA13	Promote tumor proliferation	−	[113]
DCAF^f^	BRCA^h^ (mice)		Scrg 1, Sox9, Sox10	Promote tumor proliferation	−	[73]
	STAD^k^		−	Promote tumor proliferation	−	[114]
			DCN	Promote tumor proliferation Immunotherapy resistance	−	[115]
Me CAF^g^	BRCA^h^		HSPH1, HSP90AA1	Cellular stress Glucose metabolism	EMT‐related pathways TGF‐β/KRAS/PI3K/AKT pathway	[41]
		CSF3^+^ CAF	CSF3	Glucose metabolism	Glycolysis pathway	[116]
	PAAD^i^		PLA2G2A, CRABP‐2	Protein translation Glucose metabolism	−	[117, 118]
	LUAD^l^	CTHRC1^+^ CAF	CTHRC1	Glucose metabolism	TGF‐β pathway	[119]
	CHOL^t^	Lipid metabolism	APOA2, FABP1, FABP4		−	[64, 79]
	LIHC^m^	Lipid metabolism	CD36, MIF	Immunosuppression	IL‐6/STAT‐3	[120]
	COAD^j^	POSTN^+^ CAF	POSTN	Promote tumor proliferation	Hypoxia pathway	[121]
	Melanoma	Cholesterol metabolism	CLU	Activate complement	−	[78]
Stemness CAF	BRCA^h^	CD10^+^ GPR77^+^CAF	CD10, GPR77	Maintain cancer stemness	NF‐kB pathway	[122]
	LUAD^l^	CD10^+^ GPR77^+^CAF	CD10, GPR77	Maintain cancer stemness	NF‐kB pathway	[122]
		hsCAF	IGF1	Promote tumor stemness	AMPK pathway	[123]
	COAD^j^	Netrin‐1^+^ CAF	α‐SMA	Maintain cancer stemness	JAK/STAT pathway	[124]
	HNSC^p^	CAF1	CTHRC1, COL1A1, POSTN, TPM4, MFAP2	Promote tumor stemness	PTK7–Wnt/β‐catenin pathway	[125]
Other CAF	BRCA^h^	Reticular‐like CAF	CCL21, CCL19	Promote homing of naive T cells	−	[41]
		POSTN^+^ CAF	POSTN	Promote chemotherapy resistance	−	[126]
	LIHC^m^	PF/MesCAF	Mesocortex markers	Involvement in neuroendocrine	−	[63, 64]
		Unnamed CAF	−	Secreting IDO and PGE2 Inactivate NK cells	−	[93]
	CESC^o^	Periostin^+^ CAF	Periostin	Disruption of lymphatic endothelial barrier Promotes tumor lymph node metastasis	−	[127]
	Melanoma	Pin^+^ CAF	Pin	Hyperplasia of connective tissue	−	[128]
	LUAD^l^	s3‐CAF	CXCL14, CFD, CD74, TAP2	Immunosuppression Antigen presentation	−	[61]
		s4‐CAF	STAT3, CXCL9, CCL2, CCL19, CCL21, TAP2	Immunosuppression Antigen presentation	−	[61]
		FHL2^+^ CAF	FHL2	Promote tumor proliferation	−	[129]
	HNSC^p^	CCL19^+^ CAF	CCL19	Immunosuppression	−	[96]
	PAAD^i^	EndoCAF	FAP, CD144	Promote tumor proliferation Promote migration	CD144–β‐catenin–STAT3 pathway	[130]
		Senescent CAFs	CXCL12, MIF, VEGF	Immunosuppression and angiogenesis	MIF pathway	[130]
		CXCL10^+^ CAF	CXCL10	Promote tumor proliferation	−	[131]
	COAD^j^	NOTCH3^+^ CAF	NOTCH3	Promote tumor proliferation and migration	−	[132]
		FGF19^+^ CAF	FGF19	Promote tum or migration	−	[133]
	STAD^k^	SUSD2^+^ CAF	SUSD2	Immunotherapy resistance	−	[134]
	Pan‐cancer	MMP1^+^ CAF	MMP1	Immunosuppression	−	[46]
	Mice model	SFRP2^+^ CAF	SFRP2	Decrease the effectiveness of radiotherapy	−	[135]

*Note*: a: myofibroblastic CAF, b: inflammatory CAF, c: antigen‐presenting CAF, d: vascular CAF, e: cycling CAF, f: developmental CAF, g: metabolic CAF, h: breast invasive carcinoma, i: pancreatic adenocarcinoma, j: colon adenocarcinoma, k: stomach adenocarcinoma, l: lung adenocarcinoma, m: liver hepatocellular carcinoma, n: bladder urothelial carcinoma, o: cervical squamous cell carcinoma, p: head and neck squamous cell carcinoma, q: esophageal carcinoma, r: renal cell carcinoma, s: ovarian cancer, t: cholangiocarcinoma, u: thyroid carcinoma, v: gallbladder cancer, w: oral squamous cell carcinoma, x: prostate adenocarcinoma.

### 3.2 Diverse Role of CAFs in Tumor Immunity

As an important component of tumor stroma, CAFs actively interact with immune cells in the TME. Different subtypes of CAFs have different roles in tumor immunity, contributing to the complexity of the tumor immune microenvironment. Immune‐associated CAFs can be classified as immunoactivating CAFs and immunosuppressive CAFs. In recent years, new subtypes of immunoactivating CAFs and immunosuppressive CAFs have been identified and their significance in tumor immunology attract tremendous attention.

#### 3.2.1 Immunoactivating CAFs

Immunoactivating CAFs are defined by their interactions with immune cells (such as macrophages, natural killer [NK] cells, or CTLs) and their ability to enhance immune activation. Although these CAFs are not abundant within the TME, apCAFs are a notable example. In PDAC and cholangiocarcinoma, apCAFs are characterized by high expression levels of MHC‐II genes, which endows them with antigen‐presenting capabilities that can promote immune activation to some extent [44, 79]. However, apCAFs can induce the conversion of naive CD4^+^ T cells into Tregs, thereby exerting immunosuppressive effects [35]. In early‐stage PDAC, Chen et al. [45] identified a subtype of CAFs that secretes complement components (such as C3 or C7), termed complement‐secreting CAFs, and reported that these cells significantly inhibit PDAC progression.

Additionally, ECM‐mCAFs, which highly express the immune cell‐attracting factor CXCL14, are positively correlated with overall patient survival rates in breast cancer [136, 137]. This finding suggested that ECM‐mCAFs may promote immune cell infiltration in breast cancer by secreting CXCL14, thereby influencing patient survival. Cords et al. [41] identified a specialized subset of CAFs in breast cancer that actively respond to interferon signaling and upregulate immune regulatory genes, such as CXCL9, CXCL10, CXCL11, and IDO1, in response to interferon. These CAFs have been termed interferon‐responsive CAFs, indicating that this subtype of CAF may also enhance the functionality of immune cells. Furthermore, Khaliq et al. [53] reported a link between wound‐myCAFs and high levels of T lymphocyte infiltration within tumors. Given the limited reports on immunoactivating CAFs, the discovery of additional CAFs could not only broaden the scope of CAF research but also potentially improve the efficacy of cancer immunotherapy.

#### 3.2.2 Immunosuppressive CAFs

We defined CAFs that interact with immune cells and exhibit immunosuppressive functions as immunosuppressive CAFs. iCAFs, a type of CAF commonly found in various tumors, possess potent IM capabilities. In renal cell carcinoma, the CD248^+^ CAF area subtype of iCAFs contributes to the formation of the TIME by depleting CD8^+^ T lymphocytes and recruiting M2‐type tumor‐associated macrophages (TAMs) [101]. In breast cancer, iCAFs promote T‐cell exclusion by secreting CXCL12, which binds to CXCR4 on T cells [41, 137]. Concurrently, iCAFs promote a T helper type 2 (Th2) response rather than a T helper type 1 (Th1) response in pancreatic cancer, thus mediating immunosuppression [49].

In addition to classic iCAFs, other subtypes of CAFs also possess immunosuppressive functions. In a mouse model of triple‐negative breast cancer (TNBC), PDPN^+^ CAFs were confirmed to suppress the functionality of NK cells and inhibit antibody‐dependent cellular cytotoxicity (ADCC) [136, 138], suggesting their potential as novel therapeutic targets. Furthermore, in hepatocellular carcinoma (HCC), IL‐6 derived from CAFs recruits neutrophils through the STAT3/PD‐L1 signaling pathway. This process regulates neutrophil survival and function, thereby enhancing the immune tolerance of HCC cells [92]. Additionally, HCC‐derived CAFs promote immune tolerance by inactivating NK cells through the secretion of IDO and PGE2, thereby facilitating HCC progression [93]. Zhu et al. [120] identified a CD36^+^ CAF subtype, a subset of Me CAFs, characterized by high lipid metabolism and macrophage migration inhibitory factor (MIF) expression. MIFs derived from these CD36^+^ CAFs activate myeloid‐derived suppressor cells (MDSCs) through the IL‐6/STAT‐3 pathway, promoting an immunosuppressive TME and enhancing tumor stem cell capabilities. Koncina [139] highlighted that IL‐1R1^+^ CAFs promote colorectal cancer progression and immunosuppression by inhibiting macrophages. Similarly, ECM‐myCAFs and TGF‐β‐myCAFs are enriched in colorectal tumors characterized by the presence of Tregs and exhausted CD8^+^ T cells, suggesting that these myCAF subtypes contribute to the immunosuppressive network within the TME [53]. Using scRNA‐seq, Grout et al. [60] identified two mCAF subtypes associated with T‐cell exclusion in lung cancer. One subtype (MYH11^+^ α‐SMA^+^) present in early‐stage lung cancer promotes T‐cell marginalization through the secretion of collagen IV, while the other subtype (FAP^+^ α‐SMA^+^) found in late‐stage lung cancer repels T cells through the secretion of collagen XI/XII, exerting an immunosuppressive effect. In esophageal squamous cell carcinoma, CAFs secrete WNT2 to inhibit the SOCS3/p‐JAK2/p‐STAT3 pathway, thereby suppressing CD8^+^ T‐cell activation [99].

The comprehensive understanding of CAF–immune interactions reveals both challenges and opportunities for cancer immunotherapy. Collectively, immunosuppressive CAFs interact with macrophages, NK cells, CTLs, and other immune cells to foster an immunosuppressive environment that aids in tumor immune evasion. These findings underscore the need for subtype‐specific therapeutic approaches that can selectively target immunosuppressive CAF functions while preserving or enhancing their immunoactivating counterparts. Future research should focus on deciphering the molecular switches that determine CAF polarization to develop more precise stromal modulation strategies.

## 4 Tumorigenicity of CAFs with Novel Mechanism

The tumor‐promoting capacity of CAFs extends far beyond their structural support role in the TME. Emerging research reveals that CAFs employ sophisticated molecular mechanisms to drive tumor progression, metastasis, and therapy resistance through dynamic interactions with tumor cells and immune components. This section synthesizes recent breakthroughs in understanding how CAFs orchestrate these protumorigenic processes through novel biological pathways.

### 4.1 CAFs Promote Tumor Progression and Drug Resistance

**FIGURE 2:**
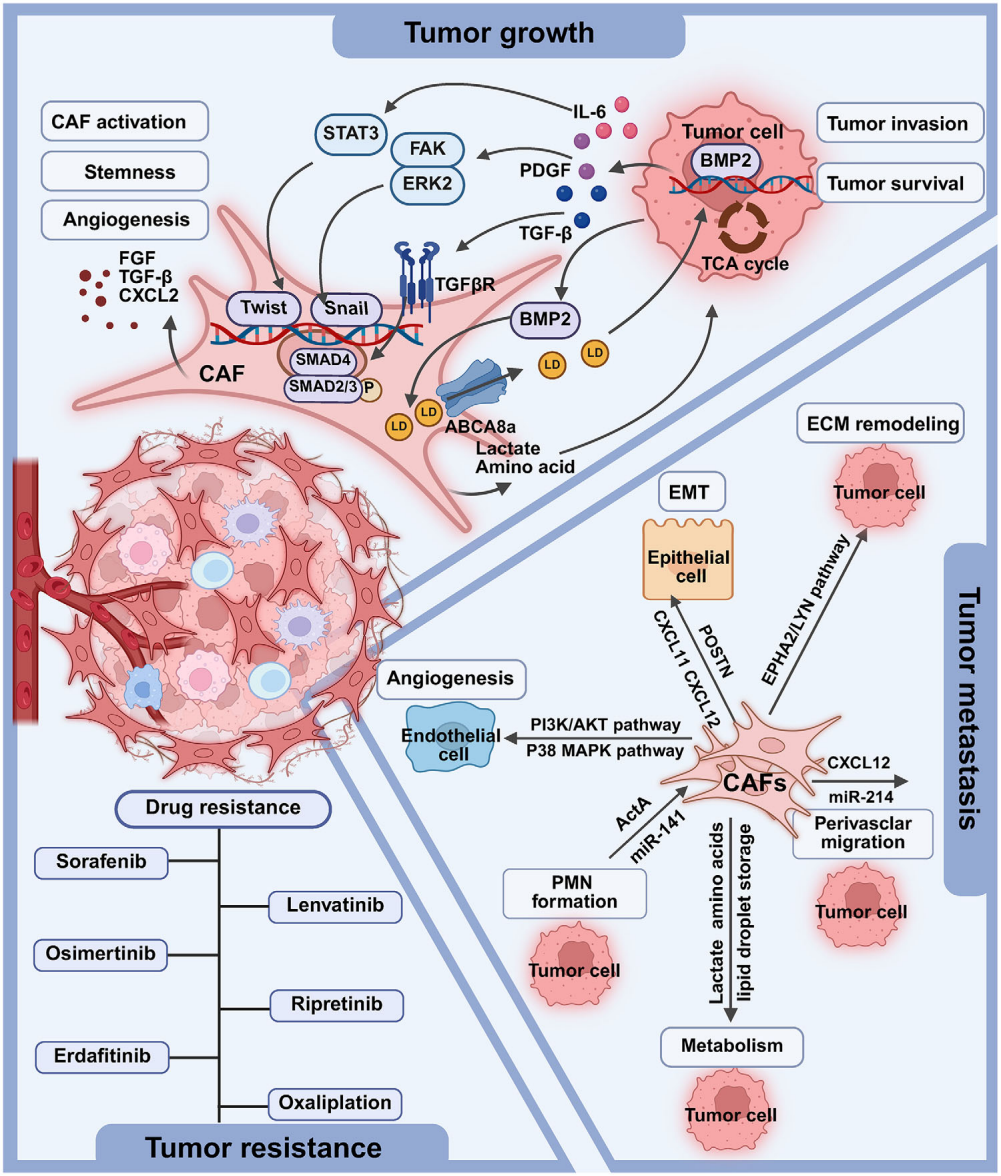
CAFs in tumor growth, metastasis, and drug resistance. Schematic illustration of the functional heterogeneity of CAFs in the TME. CAFs have been demonstrated to play several roles, including promoting tumor growth, metastasis formation, and drug resistance.

CAFs serve as master regulators of tumor malignancy, employing diverse strategies to support cancer cell survival, proliferation, and treatment evasion. The TME serves as a pivotal niche that supports tumorigenesis and metastasis at every stage. As a key component of the TME, CAFs interact with tumor cells to exert a range of tumor‐promoting functions, including facilitating tumor growth, enhancing metastasis, and mediating drug resistance (Figure 2).

#### 4.1.1 CAFs Enhance Tumor Growth

Tumor cell proliferation is a hallmark of cancer malignancy, driven by autocrine signaling and interactions with the TME that create feedback loops promoting further growth [1]. CAFs, a key component of the TME, secrete a range of cytokines—including TGF‐β, ILs (IL‐2, IL‐6), chemokines (CXCL2, CXCL5, CXCL12), and FGF7—to support tumor cell proliferation [140]. Additionally, CAFs regulate tumor cell metabolism by modulating glucose, amino acid, and lipid metabolism, providing nutrients that fuel tumor progression [1]. For example, in breast cancer, CAFs secrete exosomal lncRNA SNHG3 to accelerate glycolysis and promote tumor cell proliferation [141]. In pancreatic cancer, CAF‐derived acetate supports tumor cell survival in an acidic microenvironment by regulating the ACSS2–SP1–SAT1 axis, facilitating tumor progression [142]. Niu et al. [143] identified a lipid‐rich CAF subpopulation in SETD2‐deficient pancreatic tumors, characterized by ABCA8a expression. The loss of SETD2 activates the BMP2 signaling pathway, driving CAFs to adopt a lipid‐rich phenotype that supports tumor cell growth through lipid transfer, thus promoting pancreatic cancer progression [143]. Conversely, tumor cells can drive CAF formation and maintain their activated state, inducing CAFs to support tumor malignancy [1]. Fang et al. [144] reviewed feedback loops between tumor cells and CAFs involving TGF‐β, Hedgehog, NF‐kB, and MAPK pathways that support tumor progression. Activated CAFs can also modulate the TME to further drive tumor progression. For instance, Jiang et al. [145] identified ANO1 as a precision therapy target in gastrointestinal cancers. ANO1 inhibits ferroptosis in tumor cells via phosphatidylinositol‐3‐kinase (PI3K)–Akt signaling and activates CAFs through TGF‐β release, while suppressing CD8^+^ T cell cytotoxicity, thereby exacerbating tumor progression. In lung cancer, lactate from tumor cells promotes the nuclear translocation of NUSAP1, activating DESMIN transcription and CAFs. DESMIN^+^ CAFs release IL‐8 to induce an immunosuppressive microenvironment through TAM‐M2 polarization, further driving tumor progression [146]. The nervous system, another critical component of the TME, also plays a significant role in tumor malignancy. Although interactions between CAFs and nerves are not fully understood, recent studies in PDAC show that CAFs are sources of nerve growth factor (NGF) and axonal guidance molecules such as Slit guidance ligand 2. These molecules promote neurite growth and neural invasion, thereby supporting tumor cell proliferation [147]. Similarly, in colorectal cancer, norepinephrine induces CAFs to secrete NGF in an ADRB2‐dependent manner. NGF‐high CAFs promote colorectal cancer cell proliferation by activating the PI3K/AKT pathway [148].

#### 4.1.2 CAFs Facilitate Tumor Metastasis

Metastasis is a stepwise process and a major determinant of poor prognosis. CAFs, the most abundant stromal cells in the TME, have been systematically reviewed by Liu et al. [149] for their key regulatory mechanisms in tumor metastasis. Under the influence of signaling pathways such as TGF‐β and Wnt/β‐catenin, CAFs drive ECM remodeling by upregulating HA, promoting ECM deposition and increasing stromal stiffness, thereby facilitating tumor metastasis. CAFs secrete cytokines like CXCL11, CXCL12, CCL2, and VEGF, as well as exosomal miRNAs such as miR‐210 and miR‐135b‐5p. These molecules promote EMT and angiogenesis, supporting tumor cell survival and migration. CAFs also play a crucial role in establishing premetastatic niches (PMNs) in distant sites from the primary tumor, while tumor cells activating CAFs via exosomal miR‐141 and Activin A to induce PMN formation. Moreover, CAFs transport various metabolites via secreted vesicles to support tumor cell survival in the harsh environment of metastasis, accelerating tumor progression. CAFs also help tumor cells evade immune surveillance by inducing an immunosuppressive microenvironment, thus promoting metastasis. For instance, in PDAC, leukemia inhibitory factor secreted by tumor cells and progranulin from macrophages activate myCAFs via the JAK/STAT3 pathway. This, in turn, promotes TAM‐M2 polarization and T cell cytotoxicity suppression, leading to immune evasion and liver metastasis [150]. Therefore, CAFs play a key role in ECM remodeling, EMT promotion, angiogenesis, PMN formation, tumor metabolism regulation, and immune evasion to assist tumor metastasis [149]. EndoMT is a hallmark of lymphatic remodeling. It involves the phenotypic conversion of lymphatic endothelial cells (LECs) into spindle‐shaped, mesenchymal‐like cells with high migratory and invasive properties, emphasizing their role in tumor metastasis. Wei et al. [151] found that in cervical squamous cell carcinoma (CSCC), PAI‐1 secreted by CAFs promotes LEC endocytosis, facilitating lymphatic metastasis. Mechanistically, PAI‐1 activates AKT/ERK1/2 pathways by interacting with low‐density lipoprotein receptor‐related protein, enhancing LEC endocytosis and tumor metastasis. Neutrophil extracellular traps (NETs) are a form of neutrophil death distinct from apoptosis and necrosis.

Research has shown that NETs can reshape the TME, capturing tumor cells and promoting metastasis [152]. Li et al. [153] discovered that in colorectal cancer liver metastasis, (FGF19) secreted by tumor cells induces HSCs into iCAFs. These iCAFs promote neutrophil infiltration and NET formation via complement C5a and IL‐1β, accelerating liver metastasis. During the early stages of metastasis, the majority of tumor cells undergo oxidative stress as they detach from the local ECM. This process often results in cell death and significantly diminishes metastatic efficiency [154]. However, Zhou et al. [155] showed that CAFs participate in tumor metastasis by alleviating oxidative stress. EVs from highly metastatic lung cancer cells transport miR‐1290 into quiescent fibroblasts, leading to activate the AKT signaling pathway and induce NFs to iCAFs. These activated iCAFs exhibit increased mitochondrial autophagy and mitochondrial DNA release, conferring oxidative stress resistance and promoting high metastatic potential in lung cancer. These findings reveal novel mechanisms that promote metastasis, offering potential preventive and therapeutic strategies for lung cancer and new directions for metastasis research in other cancers. Overall, CAFs exert diverse regulatory roles in the complex process of distant tumor dissemination, highlighting their clinical translational significance of targeting CAFs. Single‐cell and transcriptomic sequencing have advanced the classification of CAFs into distinct functional subgroups. Further investigation of the dynamic changes of CAF subpopulations during tumor metastasis, as well as the cytokines and signaling pathways they are involved in, will pave the way for the development of more precise and efficient inhibitors, ultimately achieving greater breakthroughs and progress in the treatment of tumor metastasis.

#### 4.1.3 CAFs Promote Multiple Drug Resistance

Beyond fostering progression and metastasis, CAFs confer drug resistance via cytokine secretion, exosome release, and EMT induction. The ECM, a key component of the TME enriched with glycoproteins and proteoglycans, serves as a niche for cancer stem cells (CSCs) and drives drug resistance. Additionally, the ECM's dense fibrous network can impede drug penetration, contributing to physical barriers against tumor therapies [156]. Sorafenib and lenvatinib, both receptor tyrosine kinase inhibitors (TKIs), are used to inhibit the progression of HCC by targeting multiple receptor tyrosine kinases. CAFs mediate resistance to these TKIs by secreting SPP1, which activates the PI3K/AKT/mTOR pathway [157]. Under hypoxic conditions, HIF‐1α prompts tumor cells to secrete PDGF, which subsequently mediates CAF resistance to ripretinib via the PDGFR signaling pathway. This suggests that targeting CAFs in combination with ripretinib could enhance therapeutic outcomes in ovarian clear cell carcinoma [158]. The fibroblast growth factor receptor (FGFR) family regulates various cellular functions, including proliferation, differentiation, and migration. Genomic aberrations in FGFR are prevalent in metastatic urothelial cancer (mUC) [159]. Erdafitinib is a pan‐FGFR inhibitor. Hosni et al. [160] discovered that iCAF‐derived neuregulin 1 (NRG1) mediates resistance to erdafitinib in mUC via the HER3 signaling pathway. This study paves the way for therapeutic targeting of NRG1 in CAFs. Additionally, in urothelial bladder cancer, CAF‐derived exosomal miR‐146‐5p targets ARID1A and PRKAA2 in tumor cells, promoting CSC stemness and chemoresistance. These findings suggest that targeting exosomal miR‐146‐5p could be a therapeutic strategy to improve drug resistance [161]. EMT can induce chemotherapy resistance by arresting the cell cycle or altering the expression of drug transporters that facilitate the uptake of chemotherapeutic drugs [156]. In colorectal cancer, THBS2^+^ CAFs specifically secrete COL8A1, which binds to the ITGB1 receptor on tumor cells. This interaction activates the EMT process via the PI3K–AKT pathway, ultimately leading to oxaliplatin resistance in colorectal cancer [162]. In TNBC, high expression of CD146 in the tumor stroma downregulates epithelial markers, while upregulating mesenchymal markers. CD146, a unique EMT activator, significantly enhances tumor cell migration and invasion, thereby promoting tumor progression [156]. Acquired resistance is a major barrier to the efficacy of osimertinib in treating lung adenocarcinoma (LUAD). Huang et al. [163] demonstrated that CAF‐derived colony‐stimulating factor 2 (CSF2) activates the JAK2/STAT3 axis, promoting tumor cell expression of lnc‐CSRNP3. Subsequently, lnc‐CSRNP3 binds to chromodomain helicase DNA binding protein 9 (CHD9), inhibiting the phosphatase activity of serine/threonine protein phosphatase 1 catalytic subunit α (PP1α), thereby enhancing ribosome biogenesis and inducing osimertinib resistance. In summary, CAFs enhance drug resistance in tumor cells through multiple mechanisms, including secreting soluble factors, remodeling the ECM, inducing EMT, and maintaining CSC properties. Elucidating the mechanisms through which CAFs promote multidrug resistance is crucial for developing more effective anticancer therapies and offering new hope to cancer patients.

### 4.2 Mechanisms Underlying CAFs‐Mediated Immunotherapy Resistance

Cancer immunotherapy has revolutionized the field of oncology, demonstrating remarkable efficacy in prolonging the survival of cancer patients. Currently, immune‐based therapies have been adopted as first‐line treatments for many eligible cancer indications. However, inducing durable and effective responses remains limited in a significant proportion of patients [164]. Given the highly heterogeneous ecosystem of the TME, a comprehensive understanding of the mechanisms by which CAFs drive immunotherapy resistance is crucial.

#### 4.2.1 CAFs Mediate Immune Resistance by Regulating Immune Checkpoint Expression

Immunotherapy has ushered in a new era in cancer treatment, transforming the therapeutic landscape for various human malignancies. Immune checkpoint blockade (ICB) is based on inhibiting tumor‐mediated suppression of antitumor immune responses [165]. Monoclonal antibodies targeting immune checkpoint proteins such as CTLA‐4, PD‐1, and PD‐L1, have been approved by regulatory authorities for use in cancer therapy. However, CAFs have been identified as a key factor contributing to resistance to ICB therapy. CAFs can enhance the expression of PD‐L1 on tumor cells, which subsequently binds to PD‐1 on activated T cells, thereby inhibiting T cell activation signals and limiting the function of cytotoxic T cells, ultimately blocking antitumor immunity. In bladder cancer, CAF‐derived CXCL12 activates the downstream JAK2/STAT3 pathway, promoting the expression of the deubiquitinase CYLD and the accumulation of p62, thereby inhibiting the autophagic degradation of PD‐L1 and inducing immune evasion [166]. In GC, IL‐8 secreted by CAFs activates the NF‐kB pathway, upregulating PD‐L1 expression in tumor cells and attenuating the cytotoxicity of CD8^+^ T cells, thereby promoting GC progression [167]. In HCC, CAF‐derived exosomes highly express circHIF1A, which is transferred to HCC cells via exosomes. Mechanistically, circHIF1A interacts with HuR to promote PD‐L1 expression in HCC cells, inducing immune evasion [168]. Similarly, in bladder cancer (BCa), CAFs release EVs that promote PD‐L1 expression in tumor cells while inhibiting the proliferation of CD8^+^ T cells and reducing the secretion of IFN‐γ, IL‐2, and TNF‐α by CD8^+^ T cells, ultimately leading to immunotherapy resistance [169]. Autophagy is a multistep regulatory mechanism that delivers materials to lysosomes for degradation to meet nutritional needs. In addition to contributing to fibrosis, autophagy in CAFs plays a crucial role in the modulation of antitumor immune responses. Researchers found that CAF autophagy upregulates the expression of USP14 in PDAC tumor cells through IL‐6, thereby promoting elevated levels of CD274/PD‐L1 and inducing immunotherapy resistance in PDAC [170]. Koikawa et al. [128] identified a unique prolyl isomerase, Pin1, which is highly expressed in CAFs of PDAC. They demonstrated that Pin1^+^ CAFs enhance PD‐L1 expression on tumor cells, rendering immunotherapy ineffective.

**FIGURE 3:**
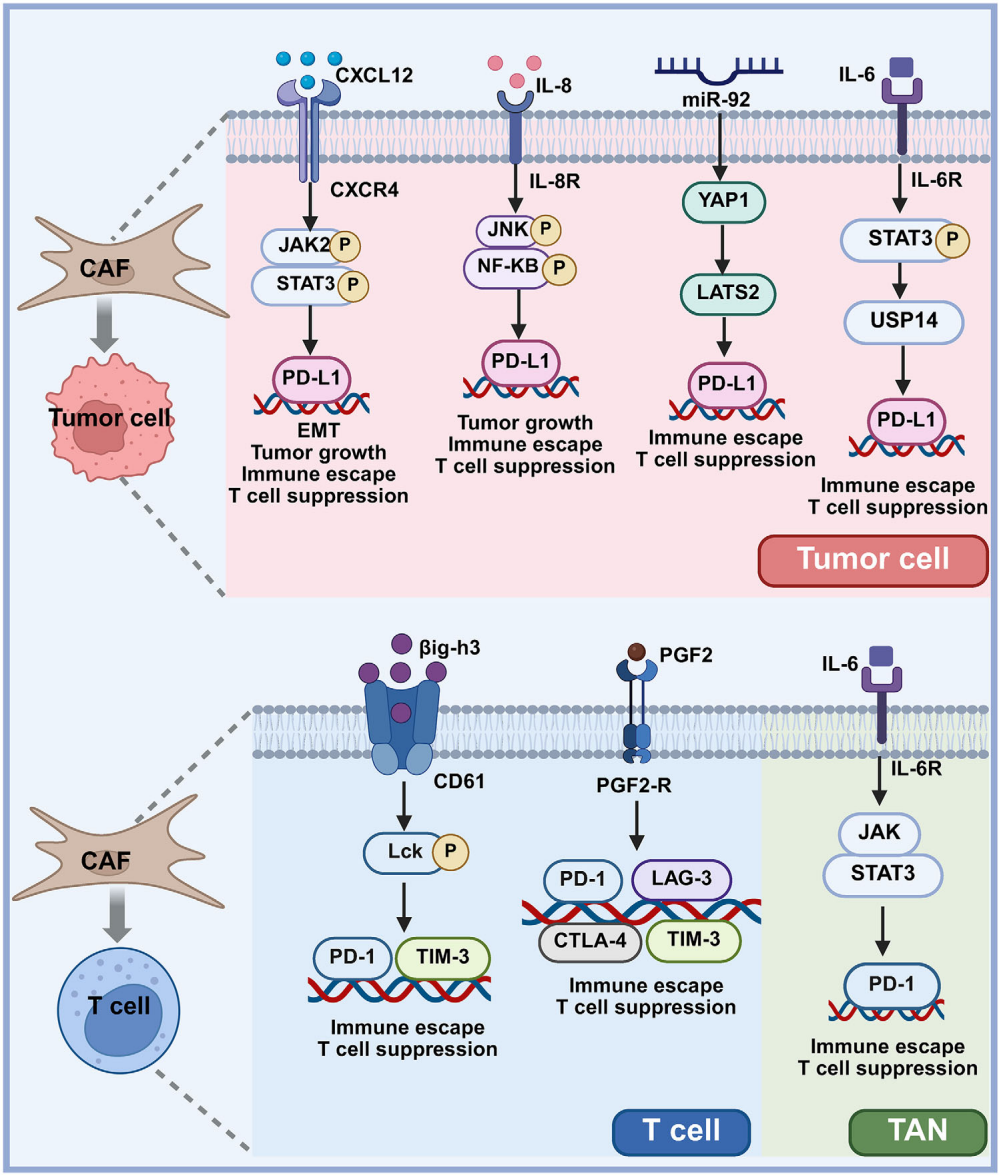
CAFs upregulate immune checkpoints on cancer cells and immune cells. CAFs promote the expression of PD‐L1 on tumor cells through several key signaling pathways: CAF‐derived CXCL12 binds to CXCR4 on tumor cells to activate the JAK/STAT3 pathway. CAFs secrete IL‐6 and IL‐8 to promote STAT3 and JNK phosphorylation, upregulating PD‐L1, and driving EMT. CAF‐secreted miR‐92 further boosts PD‐L1 via a YAP1/LATS2 axis. Concurrently, PGF2, IL‐6 and βig‐h3 enhance PD‐1, CTLA‐4, LAG‐3, and TIM‐3 on T cells and TANs, thus suppressing antitumor immunity.

Multiple studies have shown that following ICB therapy, CAFs can upregulate inhibitory checkpoint molecules on T cells as a compensatory response. For example, myCAFs from pancreatic cancer release PGE2, which increases the expression of PD‐1, CTLA‐4, TIM‐3, and LAG‐3 on CD8^+^ T cells, thereby suppressing their antitumor functions [171]. Concurrently, targeting myCAF‐secreted βig‐h3 can reduce the expression of PD‐1 and TIM‐3 on CD8^+^ T cells, thereby restoring T cell function [172]. In HCC, iCAF‐derived IL‐6 upregulates PD‐1 expression on CD8^+^ T cells and induces PD‐L1 expression on neutrophils by activating the STAT3 signaling pathway, ultimately impairing T‐cell function and inducing immune tolerance [92]. Agorku et al. [173] identified a novel immunosuppressive CAF subpopulation in colorectal cancer, termed T cell‐inhibiting CAF (TinCAF), which exhibit potent T cell‐suppressive activity. NECTIN2, a transmembrane glycoprotein with an immunoglobulin‐like domain also known as CD112 or PVRL2, has been shown to interact with multiple receptors on T cells and NK cells. The study revealed that high expression of NECTIN2 by TinCAFs suppresses T cell cytotoxicity and induces an immunosuppressive microenvironment, suggesting that NECTIN2^+^ TinCAFs may represent a novel target for ICB therapy. However, although blocking NECTIN2 can partially restore T cell function, it is not sufficient to fully restore T cell proliferation. This suggests that TinCAFs may express additional IM molecules. Future research should focus on the extent to which CAFs shape the immunosuppressive microenvironment through these molecules and the potential for targeting them to enhance the efficacy of ICB therapies (Figure 3).

#### 4.2.2 CAFs Mediate Immune Resistance by Regulating Immune Cells

**FIGURE 4:**
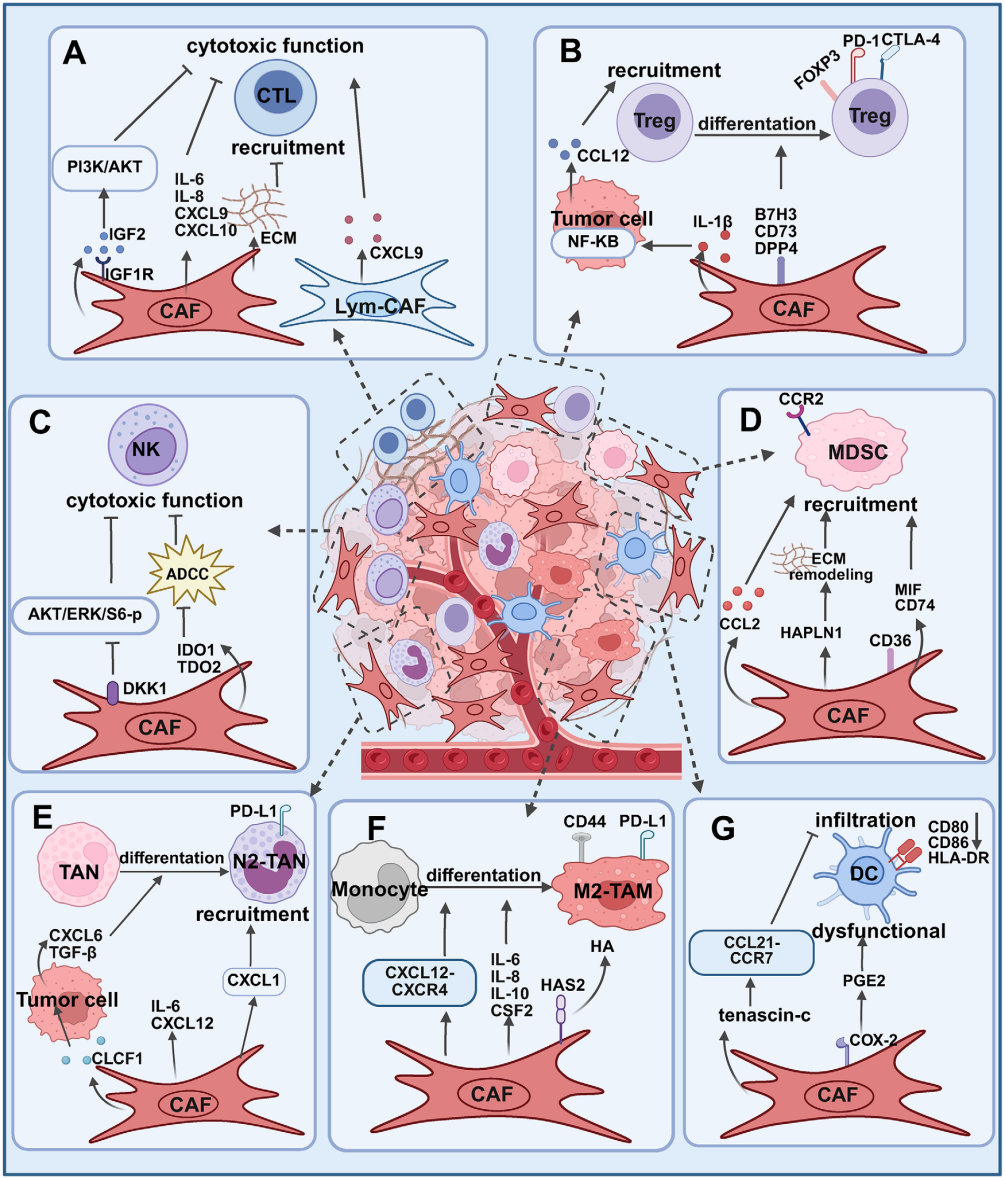
Crosstalk between CAFs and immune cells in the TME. CAFs constitute the dominant stromal population in the TME. Beyond their direct crosstalk with tumor cells that fuels tumor progression, they release many cytokines, chemokines, and other effector molecules that prompt immune cells recruitment and activation, thereby modulating the tumor immune microenvironment and inhibiting the antitumor immune response.

The interactions between CAFs and immune cells in the TME are critical for tumor progression and resistance to therapy. The key mechanisms underlying the crosstalk between CAFs and various immune cells in the TME are illustrated in Figure 4.

##### 4.2.2.1 Cytotoxic T Lymphocytes

T lymphocytes, including CTLs and Tregs, are pivotal in modulating adaptive immune responses. Extensive researches have highlighted the role of CAFs in modulating T cell activity and function [174]. Tumor‐infiltrating CTLs, also known as CD8^+^ T cells, exert their cytotoxic functions primarily by inducing tumor cell apoptosis and serve as a critical effector in antitumor immunity. Numerous studies have shown that CAFs significantly influence T cell function and immune responses. CAFs modify the ECM's physical and chemical attributes by secreting collagen, HA, and other factors, thereby restricting T cell infiltration and impacting the efficacy of immunotherapy [175]. In pancreatic cancer, TAK1^+^ CAFs secrete CXCL1 to promote an immunosuppressive microenvironment and restrict CD8^+^ T cell infiltration [176]. In breast cancer, IFN‐γ‐induced CAFs upregulate elastin microfibrillar interface protein 1 (EMILIN1), an inhibitor of TGF‐β activity, to enhance CD8^+^ T cell infiltration. Reprogramming CAFs to upregulate EMILIN1 may be a promising therapeutic strategy to improve immunotherapy efficacy [177]. Additionally, in bladder cancer, the JNK signaling pathway promotes the expression of thymic stromal lymphopoietin in CAFs, which inhibits CD8^+^ T cell cytotoxicity and effector functions, ultimately contributing to immunotherapy resistance [178]. Similarly, CAFs secrete insulin‐like growth factor 2 (IGF2), which binds to the IGF1R on CAFs to activate the PI3K/AKT pathway and lead to CXCL12 release. This ultimately reduces T cell secretion of IFN‐γ and TNF‐α, impairing CD8^+^ T cell cytotoxicity [179]. CAFs also secrete chemokines such as CXCL9, CXCL10, IL‐6, and IL‐8 to suppress T cell activity [180, 181]. However, certain CAF subtypes can enhance antitumor immunity. For instance, in prostate cancer (PCa), lymphocyte‐associated CAFs (Lym‐CAFs) exhibit an antitumor phenotype and induce CD8^+^ T cell infiltration and activation. Knocking down YAP1 in myCAFs induces their conversion to Lym‐CAFs, enhancing CD8^+^ T cell infiltration and improving the efficacy of anti‐PD‐1 therapy [182]. Similarly, Xu et al. [183] found that in HCC, FOM2^+^ CAFs boost CD8^+^ T cell infiltration via NF‐kB/p65‐mediated CCL19 expression, correlating with better anti‐PD‐1 responses.

##### 4.2.2.2 Nature Killing Cells

NK cells, as components of the innate immune system, initiate antitumor cytotoxicity in a variety of solid tumors. TGF‐β is a key factor linking CAFs and NK cells, significantly impacting NK cell immune functions. For example, in bladder cancer, TGF‐β downregulates CD16 on NK cells, dampening their cytotoxicity [184]. Gao et al. [185] demonstrated that TGF‐β can transdifferentiate NK cells into cytotoxicity‐deficient innate lymphoid cells type 1 (ILC1), facilitating immune evasion. In breast cancer, CAFs expressing Dickkopf‐1 (DKK1)—a Wnt/β‐catenin inhibitor linked to disease progression—attenuate NK cell activation and cytotoxicity by downregulating AKT/ERK/S6 phosphorylation [85]. Additionally, in GC, CAFs impair NK cell antitumor activity by inducing iron accumulation. Mechanistically, CAFs upregulate ferroportin1 and hephaestin, promoting iron uptake by NK cells. CAF‐derived follistatin‐like protein 1 (FSTL1) upregulates NCOA4 in NK cells via the DIP2A–P38 pathway, inducing iron accumulation and reducing NK cell cytotoxicity [186]. Trastuzumab, a first‐line therapy for HER2^+^ breast cancer, functions by binding to the Fcγ receptor on immune cells, triggering the release of cytotoxic factors in a process known as ADCC. Du et al. [138] showed that PDPN^+^ CAFs secrete IDO1 and TDO2, which inhibit NK cell‐mediated ADCC and promote trastuzumab resistance. Ye et al. [187] discovered that senescent myofibroblasts (senCAFs) in breast cancer limit NK cell cytotoxicity via ECM secretion, driving immune evasion. They proposed targeting senCAFs with senolytic therapies to inhibit breast cancer progression. Overall, these studies emphasize the roles of CAFs in modulating antitumor immunity and NK cell activity within the TME. However, the impact of NK cells on CAFs remains underexplored, highlighting the need for further investigation into their interplay.

##### 4.2.2.3 Dendritic Cells

Dendritic cells (DCs), key players in antigen presentation, drive T cell activation and immune responses within the TME. However, CAFs can impair DC maturation, antigen presentation, and immune responses. For instance, in an organotypic melanoma skin model, CAF‐rich culture medium downregulates the expression of MHC‐II and the costimulatory molecule CD86 on DCs, blocking their maturation [188]. In HCC, myCAFs reduce CD80, CD86, and HLA‐DR on DCs while promoting secretion of immunosuppressive cytokines such as IL‐10 and TGF‐β, facilitating immune evasion [156]. In esophageal squamous cell carcinoma (ESCC), CAF‐derived WNT2 inhibits DC differentiation through the SOCS3/p‐JAK2/p‐STAT3 signaling cascade, thereby suppressing immune responses [99]. Likewise, in breast cancer lung metastasis, Gong et al. [189] found that COX‐2^+^ CAFs drive DCs toward an immunosuppressive phenotype via PGE2, promoting immune evasion. In oral squamous cell carcinoma, tenascin‐C from CAFs inhibits DC migration through CCL21–CCR7 signaling, thereby blocking DC‐mediated immune responses [190]. Conversely, PDPN^+^ CAFs can increase the number of mature DCs, thereby inducing the formation and expansion of tertiary lymphoid structures, which enhance immune surveillance and boost antitumor immunity [191]. Thus, it is essential to further explore the regulatory mechanisms between CAF subsets and DCs.

##### 4.2.2.4 Regulatory T Cells

Tregs, an immunosuppressive CD4^+^ T cell subset marked by high Foxp3 expression, play a key role in dampening antitumor immunity. CAF‐S1, a myofibroblast subtype, enhances the migration of CD4^+^ CD25^+^ Tregs via CXCL12 secretion and promotes their differentiation into CD25^+^ Foxp3^+^ Tregs through the expression of B7H3, CD73, and dipeptidyl peptidase IV (DPP4), thereby facilitating immune evasion [89]. Kieffer et al. [38] identified a subset of CAF‐S1, termed ECM‐myCAFs, that contribute to immunotherapy resistance in breast cancer. ECM‐myCAFs not only increase the number of Foxp3^+^ Tregs but also upregulate PD‐1 and CTLA‐4 expression on their surface, highlighting specific CAF‐S1 clusters as key drivers of primary resistance to immunotherapy. The influence of apCAFs on Treg cells can either suppress or promote immunity, depending on the context of the TME. For instance, when cocultured with naïve CD4^+^ T cells, apCAFs can induce their differentiation into Foxp3^+^ Tregs in an antigen‐specific manner, thereby facilitating immune evasion by PDAC cells [35]. In head and neck squamous cell carcinoma (HNSCC), tumor cells secrete MIF to activate the JAK/STAT3 pathway in myeloid cells, which in turn induces the formation of apCAFs. These activated apCAFs then modulate the CD4^+^ /CD8^+^ T cell ratio to promote tumor immunosuppression [192]. In contrast, in NSCLC, apCAFs stimulate effector CD4^+^ T cells through the T cell receptor and sustain T cell survival via the C1q–C1qbp axis, thereby promoting MHC‐II‐dependent antitumor immunity [193]. The immunostimulatory properties of apCAFs may stem from their origin as alveolar type II cells, which are capable of presenting viral antigens [194]. Thus, the diverse cellular origins of apCAFs may account for their distinct immunological roles in tumors. Additionally, CAFs‐derived IL‐1β activates the NF‐kB pathway in tumor cells, driving the release of CCL22 and thereby recruiting Tregs in head and neck cancer [195]. Similarly, in breast cancer, iCAFs recruit Tregs and induce an immunosuppressive microenvironment through the CXCL12/CXCR4 axis, ultimately contributing to resistance to anti‐PD‐1 therapy [196].

##### 4.2.2.5 Bone Marrow‐Derived MSCs

MDSCs are a key immunosuppressive component of the TME. In a study on resistance to ICB immunotherapy (pembrolizumab) in metastatic GC, FAP^+^ CAFs were found to correlate positively with MDSCs in tumor tissues, mediating an immunosuppressive barrier through enhanced MDSC infiltration [197]. In HCC, CD36^+^ CAFs with high lipid metabolism promote the recruitment of CD33^+^ MDSCs via a mechanism dependent on MIF and CD74, fostering an immunosuppressive environment [120]. Similarly, in bladder cancer, ICAM1^+^ iCAFs recruit MDSCs by secreting CCL2, which binds to CCR2 on MDSCs, fostering an immunosuppressive microenvironment. Biglycan, a biomarker predictive of immunotherapy response, was found to be secreted by CAFs in TNBC. It promotes MDSC recruitment and suppresses T cell activity, thereby driving TNBC progression [198]. Moreover, the role of CAFs in ECM remodeling not only facilitates tumor metastasis but also induces immunosuppression by promoting MDSC aggregation. In a melanoma mouse model, the degradation of hyaluronan and proteoglycan link protein 1 secreted by CAFs significantly altered ECM structure, including increased collagen contraction and fiber alignment. This process promotes MDSC aggregation, suppresses T cell cytotoxicity, and impairs the efficacy of immunotherapy [199]. Collectively, these studies highlight the intimate crosstalk between CAFs and MDSCs in modulating the immunosuppressive microenvironment.

##### 4.2.2.6 Tumor‐Associated Macrophages

Given the abundance of TAMs in CAF‐rich tumor stroma suggests a strong interplay between these cell types. In HCC, researchers have identified a spatial correlation between POSTN^+^ CAFs and SPP1^+^ TAMs. POSTN^+^ CAFs recruit SPP1^+^ TAMs to form an immunosuppressive barrier, restricting T cell infiltration and promoting resistance to immunotherapy [200]. In anti‐PD‐1‐resistant TNBC patients, Timperi et al. [201] identified a significant expansion of STAB1^+^ TREM2^high^ lipid‐associated macrophages (LAMs). The study revealed that iCAFs drive monocytes to differentiate into LAMs through the CXCL12‐CXCR4 axis, suppressing T cell activation and proliferation and thus inducing immunosuppression. In breast cancer, anti‐PD‐1 pembrolizumab therapy upregulates chitinase‐3‐Like‐1 (CHI3L1) in iCAFs, a known regulator of M2‐TAM polarization. Thus, anti‐PD‐1 treatment may enhance iCAF‐induced M2 macrophage formation, contributing to immunotherapy resistance [196]. In TNBC, a specific subset of AKAP12^+^ CAFs has been linked to immunotherapy response. These CAFs secrete IL‐34, which binds to the CSF receptor (CSF1R) on TAMs to drive M2 polarization and foster an immunosuppressive environment [202]. CAFs can also stimulate M2 polarization through the release of various cytokines, including IL‐6, IL‐8, IL‐10, and CSF2 [146]. Moreover, M2‐TAMs induced by CAFs can upregulate PD‐L1 expression and reduce phagocytosis of tumor cells, thereby promoting tumor progression [174]. For instance, Yang et al. [203] found that in colorectal cancer, a high‐fat diet induces CAFs to upregulate hyaluronan synthase 2. The resulting hyaluronan binds to CD44 on M2‐TAMs, ultimately promoting PD‐L1 upregulation and diminishing the efficacy of anti‐PD‐1 immunotherapy. Collectively, CAFs recruit TAMs to form an immunosuppressive barrier and drive M2 polarization, thereby facilitating immune evasion and dampening adaptive immune responses. However, the underlying mechanisms remain to be fully elucidated.

##### 4.2.2.7 Tumor‐Associated Neutrophils

Similar to most immune cells in the TME, tumor‐associated neutrophils (TANs) exhibit diverse phenotypes and functional heterogeneity, significantly impacting antitumor immunity. In adenosquamous PDAC, IL‐1α‐dependent iCAFs promote TAN recruitment via CXCL1 signaling while reducing T cell infiltration, thereby inducing immune evasion [204]. Additionally, IL‐1 released by PDAC tumor cells can bind to the IL‐1 receptor type 1 on CAFs, activating downstream signaling pathways and recruiting TANs [205]. CAFs can influence TAN polarization through the secretion of various cytokines. For instance, in HCC, cardiotrophin‐like cytokine factor 1 from CAFs promotes the expression of CXCL6 and TGF‐β in tumor cells, driving N2 polarization of TANs and facilitating HCC progression [206]. CAF‐derived IL‐6 activates the STAT6 pathway in TANs, increasing PD‐L1 expression and promoting immunosuppression in HCC [92]. Similarly, in HCC, CAFs enhance TAN migration via CXCL12 and boost PD‐L1 on TANs through the IL‐6–JAK–STAT3 pathway, modulating antitumor immunity [19].

Collectively, the interactions between CAFs and immune cells have been identified as a key driver of tumor progression. Elucidating the complex mechanisms underlying these interactions may pave the way for novel targeted immunotherapies.

#### 4.2.3 CAF Metabolites‐Mediated Immune Resistance

**FIGURE 5:**
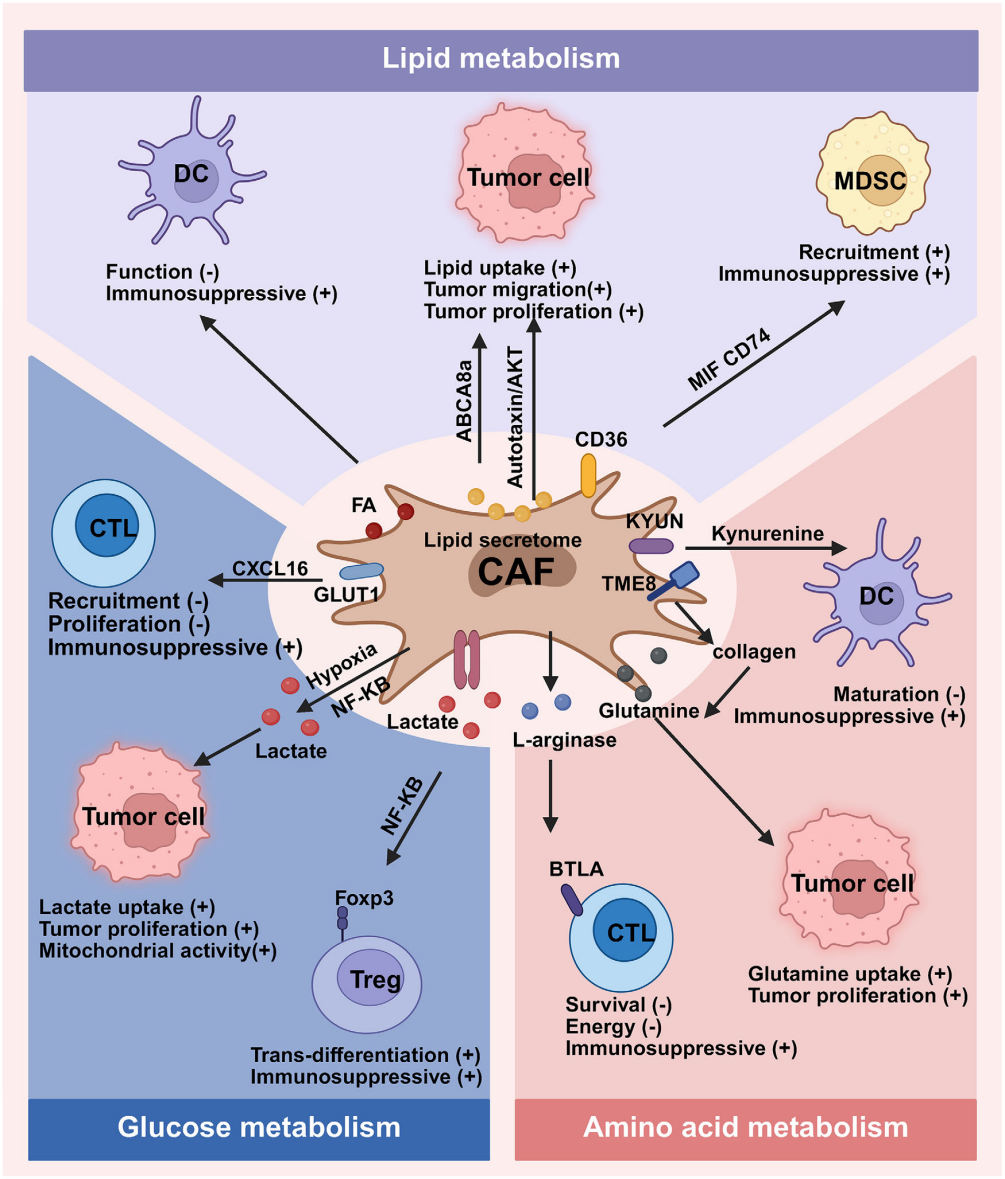
CAF metabolites‐mediated immune resistance. CAFs, critical components of the tumor microenvironment, reprogram their metabolism to nourish tumor cells, enhancing their adaptation to nutrient‐deficient conditions and influencing immune responses by modulating immune cells. The schematic illustrates that CAF metabolic reprogramming regulates antitumor immunity including glucose metabolism, amino acid metabolism, and lipid metabolism.

The TME is often hypoxic and nutrient‐poor, prompting tumor cells to alter their metabolism to adapt. CAFs, key stromal cells in the TME, show an activated phenotype and significant epigenetic reprogramming compared with NFs. This metabolic interplay with tumor cells boosts tumor cell proliferation and survival, influencing tumor growth [143] (Figure 5).

##### 4.2.3.1 Glucose

Glucose is the primary energy source for most cells and is central to cell proliferation and survival. Under hypoxic conditions, tumor cells primarily rely on glycolysis for glucose metabolism (the Warburg effect) to promote tumor progression [207]. Pavlides et al. [208] proposed the “reverse Warburg effect, ” where tumor cells induce glycolysis in CAFs. The resulting metabolites, lactate and pyruvate, nourish tumor cells, representing a key mechanism for tumor progression. In response to migration‐stimulating factor, CAFs enhance glycolysis and lactate release through NF‐kB signaling [195]. In PCa, CAFs transfer lactate to tumor cells and activate peroxisome proliferator‐activated receptor gamma coactivator 1‐alpha, enhancing mitochondrial quality and function to drive tumor progression. Similarly, in breast cancer, lactate and pyruvate produced by glycolysis in hypoxia‐induced CAFs support tumor cell growth [209]. Lactate released by CAFs not only fuels tumor cells but also drives tumor immunosuppression by modulating immune cells. Strating et al. [210] established a coculture model of colorectal cancer organoids with CAFs. Single‐cell sequencing revealed that CAFs upregulate glycolysis under coculture conditions, and the resulting lactate effectively inhibits T cell proliferation. Comito et al. [211] demonstrated that lactate released by CAFs can induce the deacetylation of T‐β transcription factors, reducing Th1 cells, and activate lactate‐dependent NF‐kB, promoting the conversion of CD4^+^ naive T cells into Foxp3^+^ Treg cells, thus fostering an immunosuppressive microenvironment and aggravating immunotherapy resistance. Additionally, in soft tissue sarcomas, a subset of glycolysis‐associated CAFs, termed glycolytic CAFs (GlyCAFs), has been identified. These GlyCAFs upregulate the expression of GLUT1‐dependent CXCL16, which prevents CD8^+^ T cells from contacting and infiltrating tumor cells, leading to immune evasion and resistance to immunotherapy [212]. In pancreatic cancer, MME^+^ CAFs exhibit heightened glycolytic activity, increasing tumor burden by recruiting neutrophils and Tregs while suppressing CD8^+^ T, NK, and DC cell infiltration, thus fostering an immunosuppressive environment that impairs immunotherapy effect [213].

##### 4.2.3.2 Amino Acid

Amino acids represent indispensable nutrients required for tumor growth, with increasing studies indicating that CAFs synthesize these critical nutrients for tumor cells via the TCA cycle [214]. The cycle's continuity requires carbon intermediates and glutamine serves as a key source of carbon and nitrogen [215]. In PCa, glutamine secreted by CAFs sustains mitochondrial bioenergetics and promotes tumor progression [216]. In melanoma and colorectal cancer models, TEM8^+^ CAFs convert collagen to glutamine to sustain tumor cells in nutrient‐deprived conditions [217]. Additionally, tumor cells can promote CAF autophagy to recycle nutrients from degraded organelles, meeting their own energy demands to sustain their malignant proliferation. For instance, tumor cells induce CAF autophagy in PDAC, leading to the secretion of nonessential amino acids, particularly alanine, to fuel the TCA cycle and support tumor progression [218]. Furthermore, amino acid metabolism in CAFs can modulate immune cells, leading to immunosuppression. For instance, adequate levels of arginine in the TME are crucial for maintaining T cell survival and activity. Researchers have found that in pancreatic cancer patients, myCAFs often express high levels of arginase II (Arg 2), particularly in hypoxic tumor regions enriched with HIF‐1α, which correlates with poor clinical outcomes. Mechanistically, Arg 2 degrades arginine into ornithine, depleting arginine in the TME and thereby suppressing T cell‐mediated antitumor immunity [6]. Melanoma‐associated fibroblasts secrete active l‐arginase, which upregulates BTLA—a potent negative regulator of T cell activity—on CD8^+^ T cells, leading to T cell dysfunction [219]. Arginine depletion can also induce the accumulation of MDSCs, thereby impairing antitumor immunity [220]. Similarly, tryptophan, an essential amino acid for T cell proliferation, is critical for T cell survival and activation, as well as for the differentiation and maturation of DCs. Depletion of tryptophan inhibits these processes and induces immune evasion by tumor cells [6]. Ravi et al. [221] developed an organotypic 3D breast TME‐on‐a‐chip model, revealing that CAFs promote tryptophan metabolism and immune evasion. KYNU is a key enzyme in the kynurenine pathway of tryptophan catabolism. CAFs upregulate KYNU, producing immunosuppressive metabolites that drive tumor progression.

##### 4.2.3.3 Lipid

In the TME, CAFs synthesize and secrete lipids and bioactive lipid signaling molecules that play crucial roles in tumor proliferation, progression, and immune responses. PSCs secrete abundant lipids during their conversion to CAFs, promoting PDAC cell proliferation via the autotaxin–LPA–AKT axis [209]. Niu et al. [143] identified a population of lipid‐rich CAFs in SETD2‐deficient pancreatic tumors, where the lipogenic pathway was induced to promote lipid synthesis. In turn, these lipid‐rich CAFs supply lipids to tumor cells via the ABCA8a transporter, enhancing tumor cell stemness. Furthermore, CAF‐mediated lipid metabolism influences the function of immune cells, driving tumor immune suppression. Zhu et al. [120] have identified a subset of lipogenic CAFs (lpmCAFs) that highly express CD36. These CD36^+^ CAFs upregulate MIF through the lipid peroxidation/p38/CEBPs axis and promote the recruitment of MDSCs in a MIF‐ and CD74‐dependent manner, inducing immune evasion and resistance to immunotherapy in HCC. Extensive in vivo and in vitro experiments confirm that CAFs release lipids into the TME, leading to lipid accumulation. Excessive lipids could impair the functions of immune cells [222]. For instance, the accumulation of excessive free fatty acids (FA) weakens the ability of DCs to process and present antigens [223]. One study showed that activated CD8^+^ T cells experience functional decline in hypoxic and nutrient‐deprived environments. The PPARα agonist fenofibrate enhances FA catabolism, boosting the cytotoxic function of CD8^+^ T cells. It significantly enhances antitumor efficacy when combined with PD‐1 blockade in vivo. Conversely, excessive lipid uptake by NK cells can suppress their cytotoxicity, ultimately inducing resistance to tumor immunotherapy [224].

##### 4.2.3.4 Nucleotide

Adenosine, an immunosuppressive metabolite, is secreted by CAFs under hypoxia via CD39 and CD73 ectonucleotidases [225]. High levels of extracellular adenosine interact with the adenosine A2A receptor on T cells, leading to cyclic AMP accumulation, which inhibits T cell proliferation and cytotoxic function and impairs NK cell activity and antitumor function. Preclinical studies in HNSCC mouse models demonstrated that the A2AR antagonist SCH58261 significantly enhances CD8^+^ T‐cell‐mediated antitumor responses, suggesting the potential of A2AR pathway inhibition to improve immunotherapy efficacy [226].

These findings indicate that CAFs exhibit a dual function in nutrient‐deprived TMEs, sustaining tumor cell viability through nutrient supply while simultaneously shaping an immunosuppressive landscape via the modulation of immune cell activity. Insights into the mechanisms by which CAF metabolic reprogramming regulates antitumor immunity may reveal novel metabolic targets for improving immunotherapy effect and overcoming immunotherapy resistance.

## 5 Emerging Technologies and Models in CAF Research

The study of CAFs has entered a transformative phase, driven by groundbreaking technological innovations that are reshaping our understanding of stromal biology in cancer. From advanced cellular models to cutting‐edge omics and computational approaches, these emerging tools provide unprecedented resolution to dissect CAF heterogeneity, function, and therapeutic vulnerabilities. This section highlights the most impactful technological advancements that are propelling CAF research forward while critically examining their current limitations and future potential.

### 5.1 Primary Cell Isolation and Differentiation Approaches

Current methodologies for investigating CAFs encompass isolation and differentiation through primary cell culture systems, as well as induction of CAFs from various precursor cell populations. PSCs in PDAC demonstrate the capacity to differentiate into CAFs, thereby driving stromal proliferation and influencing disease progression [32]. Furthermore, mesothelial cells in pancreatic cancer malignancies can be experimentally induced to transform into antigen‐presenting CAFs through stimulation with TGF‐β and IL‐1, providing a valuable model system for mechanistic investigations [35].

### 5.2 Genetically Engineered Animal Model Systems

Genetically modified murine models serve as indispensable tools for elucidating CAF biology. The KPC mouse model, extensively utilized in PDAC research, faithfully recapitulates spontaneous tumorigenesis while maintaining an intact immune microenvironment, thereby offering an exceptional platform for examining stromal–immune system cross‐talk [227]. Zhang et al. [135] employed clustered regularly interspaced short palindromic repeats (CRISPR)‐mediated genome‐wide editing to identify molecular determinants influencing the abscopal effect during radioimmunotherapy, discovering that CAFs exhibiting elevated secreted frizzled‐related protein 2 expression impair CD8‐positive T lymphocyte infiltration into nonirradiated tumor regions, consequently modulating distant therapeutic responses. To mechanistically interrogate this population, investigators generated fibroblast‐specific secreted frizzled‐related protein 2 knockout animals, establishing this factor as a promising target for enhancing radio‐immunotherapeutic efficacy.

### 5.3 Three‐Dimensional in Vitro Culture Platforms

Advanced in vitro modeling systems incorporate both two‐dimensional and three‐dimensional coculture configurations alongside patient‐derived organoid technologies. Investigators have developed an innovative three‐dimensional triculture methodology employing alginate microencapsulation within bioreactor systems, enabling coculture of non‐small cell lung carcinoma spheroids with CAFs and monocytic cells to reconstitute immunosuppressive tumor microenvironmental conditions. This platform facilitates analysis of cytokine and chemokine accumulation alongside ECM remodeling, while simultaneously permitting evaluation of cellular responses to diverse chemotherapeutic and immunotherapeutic interventions [228]. Schuth and collaborators established three‐dimensional cocultures incorporating primary PDAC organoids with patient‐derived CAFs, implementing image‐based high‐content screening to delineate stromal‐mediated chemoresistance mechanisms [229]. Such high‐throughput three‐dimensional culture systems demonstrate considerable potential for predicting clinical drug responses and facilitating personalized therapeutic strategies.

### 5.4 Single‐Cell and Spatial Transcriptomic Technologies

Contemporary molecular profiling technologies including scRNA‐seq and IMC have enabled comprehensive characterization of CAF heterogeneity, identifying distinct subpopulations including myofibroblastic, inflammatory, and antigen‐presenting variants. While these cutting‐edge technologies have significantly advanced our understanding of CAF biology, it is important to acknowledge their inherent methodological limitations. scRNA‐seq, despite its power in resolving cellular heterogeneity, may introduce biases during sample processing (e.g., cell viability and dissociation artifacts), sequencing depth variations that affect rare transcript detection, and analytical challenges in clustering and batch effect correction—all of which could influence the accuracy of CAF subtype classification and functional interpretation. Similarly, spatial transcriptomic platforms, while providing unprecedented insights into cellular microenvironments, face technical constraints including limited sensitivity for low‐abundance transcripts and spatial resolution trade‐offs that are dependent on the detection method. Next‐generation spatial transcriptomic platforms such as MERSCOPE [230], CosMx [231], and Xenium [47] permit simultaneous detection of hundreds to thousands of transcripts at single‐cell and subcellular resolution, revolutionizing investigation of stromal spatial organization within tumor ecosystems [61]. Future technological advancements addressing these limitations—such as improved single‐cell isolation protocols, higher‐throughput multiomics integration, and computational algorithms for spatial data deconvolution—will further refine CAF characterization. Liu et al. [61] employed MERSCOPE and CosMx technologies to perform extensive spatial transcriptomic mapping across eight malignancy types, identifying four spatially distinct CAF subtypes exhibiting unique organizational patterns, cellular neighborhood compositions, interaction networks, and transcriptional profiles. Similarly, Chang et al. [232] utilized Xenium in Situ and TF‐seq FISH platforms to generate single‐cell resolution spatial atlases, reconstructing the evolutionary progression of esophageal squamous cell carcinoma. These studies exemplify the transformative potential of spatial technologies while highlighting the need for methodological optimization to fully capture the complexity of CAF biology in TMEs.

### 5.5 Machine Learning and Artificial Intelligence Applications

Machine learning methodologies involve construction of predictive algorithms through training on annotated datasets, encompassing feature extraction, pattern recognition, and outcome prediction without explicit programming. These approaches are increasingly employed for data classification, dimensionality reduction, and analytical optimization in cancer research [233]. Li et al. [234] developed a CAF‐centric predictive framework for bladder cancer progression and immunotherapy response assessment, deriving a fibroblast‐related gene expression signature from large‐scale scRNA‐seq datasets that correlates with adverse clinical outcomes. The characteristic spindle‐shaped, polygonal, and stellate morphological features of CAFs provide distinctive identification criteria for artificial intelligence‐based image analysis systems. Shen et al. [235] achieved 93% diagnostic accuracy in CAF recognition through integration of Faster R‐CNN deep learning architectures with fluorescence microscopy, significantly enhancing pathological evaluation capabilities. These tools are beginning to reveal previously unrecognized CAF states and functional associations that could inform therapeutic strategies.

The continued development and implementation of these advanced investigative approaches have substantially expanded our understanding of CAF pathophysiology within malignant microenvironments, while simultaneously laying the critical foundation for therapeutic development. The integration of multiomic spatial profiling with artificial intelligence‐driven analytical pipelines represents a particularly promising avenue for future research, though current technological limitations necessitate ongoing methodological refinement to fully realize this potential.

## 6 Therapeutic Implications of CAFs

The growing recognition of CAFs as central orchestrators of tumor progression and therapy resistance has positioned them as compelling therapeutic targets in modern oncology. This section systematically examines the most promising strategies for targeting CAFs, from molecular interventions to clinical translation, while critically evaluating both the transformative potential and inherent challenges of stromal‐directed therapies.

### 6.1 Strategies to Target CAFs for Cancer Therapy

Two complementary therapeutic paradigms have emerged for CAF‐directed interventions: disrupting their activation and neutralizing their tumor‐promoting secretions. These approaches aim to dismantle the supportive niche that CAFs create for tumors while preserving their physiological functions in tissue homeostasis.

#### 6.1.1 Inhibiting CAF Activation and Recruitment

Targeting the molecular drivers of CAF activation has emerged as a pivotal strategy to disrupt tumor‐stroma crosstalk. TGF‐β signaling plays a central role in reprogramming MSCs and fibroblasts into CAFs through pathways such as JAK/STAT3 and Smad. Inhibitors like losartan and galunisertib have demonstrated efficacy in attenuating CAF activation [236, 237, 238]. Beyond TGF‐β blockade, suppressing NOX4, a key enzyme in fibroblast‐to‐CAF transformation, via inhibitors such as GKT reverses immunosuppressive CAF phenotypes and restores CD8^+^ T‐cell functionality [239]. Nanotechnology further enhances precision; for instance, Freag et al. [220] engineered nanoliposomes to simultaneously inhibit exosome‐driven fibroblast activation and dysregulated FGFR/β‐catenin signaling, synergizing with anti‐PD‐L1 therapy to overcome immune evasion. However, translating these preclinical successes into clinical applications remains challenging. Key hurdles include the redundancy of CAF‐activating pathways, stromal adaptation to targeted therapies, and the risk of disrupting homeostatic fibroblast functions in normal tissues. For example, systemic TGF‐β inhibition may lead to cardiac toxicity or paradoxical tumor promotion due to its context‐dependent roles. Future efforts should focus on combinatorial approaches that exploit synthetic lethality in CAF signaling networks while minimizing off‐target effects.

#### 6.1.2 Targeting CAF Derivatives

As pivotal orchestrators within the TME, CAFs establish a pathological ecological network that supports tumor progression through the secretion of cytokines such as IL‐6, TGF‐β, and CXCL12. Studies demonstrate that CAF‐derived IL‐6 activates the STAT3 signaling pathway to induce the expression of stemness‐associated genes such as SOX2 and NANOG, thereby promoting chemotherapy resistance [240]. Meanwhile, TGF‐β not only drives EMT but also recruits immunosuppressive Tregs and MDSCs to establish an immune‐suppressive barrier. Targeting CAF‐derived cytokines has emerged as a research priority for remodeling the TME [241]. Therapeutically, monoclonal antibodies such as tocilizumab (targeting IL‐6 receptor) have been shown to significantly reduce hepatic metastatic burden in colorectal cancer models [242]. Bifunctional fusion proteins targeting the TGF‐β pathway, including bintrafusp alfa (dual‐targeting TGF‐β and PD‐L1), demonstrated synergistic regulation of CAF density and CD8^+^ T‐cell infiltration in phase II clinical trials. Small‐molecule inhibitors like galunisertib (a TGF‐β receptor type I inhibitor) can reverse CAF‐mediated stromal desmoplasia in pancreatic cancer by blocking SMAD2/3 phosphorylation, thereby enhancing gemcitabine penetration [243].

However, these approaches face dual challenges: First, CAF heterogeneity drives spatiotemporal variations in cytokine secretion profiles such as inflammatory CAFs overexpress IL‐6, while myCAFs predominantly secrete TGF‐β, rendering monotherapies vulnerable to compensatory mechanisms. Second, systemic cytokine inhibition risks dose‐limiting toxicities, such as immune‐metabolic dysregulation caused by IL‐6 antagonism. Recent advances address these issues through single‐cell transcriptomic identification of FAP^+^ PDGFRβ^+^ CAF subpopulations specifically orchestrating the CXCL12/IL‐8 signaling axis, offering novel subtype‐selective therapeutic targets [244]. Biomimetic nanoparticle delivery systems (e.g., CAF membrane‐coated liposomes) enable precise delivery of TGF‐β siRNA to the TME, achieving synergistic CAF reprogramming and cytokine suppression in breast cancer models. Future research must integrate multiomics to decode the 3D interaction network among CAFs, immune cells, and tumor cells, while advancing dynamic‐responsive smart drug delivery systems for spatiotemporally precise modulation of cytokine microenvironments. These innovations will be critical to overcoming TME‐mediated therapeutic resistance and advancing transformative cancer treatment strategies.

### 6.2 Targeting CAFs to Overcome Immunotherapy Resistance

A growing amount of researches suggests that targeting CAFs could significantly enhance the efficacy of immunotherapy and potentially reverse immune resistance. The advent of scRNA‐seq technology has facilitated a deeper understanding of the heterogeneity within CAF populations [245]. Several strategies have been proposed to overcome immunotherapy resistance, including the selective eradicating immunosuppressive CAF subsets, reprogramming of CAFs, and disrupting the interactions between CAFs and immunosuppressive TME.

#### 6.2.1 Eliminating Immunosuppressive CAFs

Strategies to target CAFs are primarily focused on the direct elimination of CAFs by targeting their surface markers, such as α‐SMA, FAP, and PDGFR‐β [236, 237]. While these approaches hold promise, their clinical translation requires careful consideration of CAF heterogeneity. A critical limitation in this field is the lack of truly tumor‐specific CAF markers, as most targets (e.g., FAP, α‐SMA) are also expressed in physiologic wound healing or normal stromal compartments. This fundamental biological constraint explains why many CAF‐directed therapies have shown narrow therapeutic windows in early‐phase trials. For instance, Jenkins et al. [246] demonstrated that targeting α‐SMA^+^ CAFs enhances ICB sensitivity in breast cancer by improving CD8^+^ T‐cell positioning. However, α‐SMA is also expressed in nonmalignant stromal cells, raising concerns about off‐target effects. This underscores the need for more specific biomarkers to distinguish tumor‐promoting CAF subsets from those critical for tissue homeostasis. The development of FAP‐based vaccines and FAP‐CAR‐T cells represents a significant advance in stromal targeting [120, 143, 236]. Preclinical studies show that FAP‐CAR‐T cells disrupt stromal barriers and overcome immunotherapy resistance, but clinical trials have revealed challenges, including limited persistence of CAR‐T cells in fibrotic TMEs and systemic toxicities due to FAP expression in NFs. Future therapeutic strategies could explore multiple approaches. One direction involves engineering hypoxia‐responsive CAR‐T cells to confine their activity within tumor niches. Another approach targets combinatorial markers like FAP^+^ CD142^+^ to enhance treatment specificity. Additionally, CAF‐targeted bispecific antibodies could be developed to preferentially engage T cells in the TME. These complementary strategies may synergistically improve therapeutic outcomes.

Li et al. [247] identified a role for histone deacetylase 6 (HDAC6) in iCAFs in programming the immunosuppressive TME, where reducing HDAC6 in CAFs can alter the immunosuppressive breast cancer microenvironment by inhibiting MDSC and Treg recruitment and modulating macrophage phenotypes. Abexinostat, a potent oral HDAC inhibitor, may restore sensitivity to immunotherapy [248]. While abexinostat exhibits potential in restoring immunotherapy sensitivity [248], HDAC inhibitors often exhibit pleiotropic effects. The dual role of HDAC6 in CAF activation and immune regulation suggests that selective HDAC6 inhibitors, rather than pan‐HDAC inhibitors, may offer a more favorable therapeutic window. Combining HDAC6‐targeted therapy with checkpoint inhibitors could exploit synergistic effects on both CAF reprogramming and T‐cell activation.

In pancreatic cancer, the coexistence of “tumor‐promoting” FAP^+^ CAFs and “tumor‐suppressing”α‐SMA^+^ CAFs highlights the complexity of CAF‐targeted therapies. McAndrews et al. [249] proposed selective elimination of FAP^+^ CAFs while preserving α‐SMA^+^ subsets—a strategy that improved survival in preclinical models. However, translating this to clinical practice necessitates reliable methods to distinguish these subsets in human tumors, such as spatial transcriptomics or multiplexed imaging. Zhang et al. [166] showed that inhibiting CAF autophagy by deleting Atg5 in mCAFs can restore PD‐L1 expression in both CAFs and TILs. This alteration converts the TME from type I and IV to type II, thereby amplifying the efficacy of ICB and gemcitabine [102, 220]. While this approach amplifies ICB efficacy, autophagy is a survival mechanism for both tumor and stromal cells. Systemic autophagy inhibition (e.g., hydroxychloroquine) may inadvertently promote tumor cell resistance. A more refined strategy could involve localized delivery of autophagy inhibitors or the combination of these inhibitors with stromal‐targeted nanoparticles to minimize off‐tumor effects.

#### 6.2.2 Reprogramming Immunosuppressive CAFs

Cancer cell reprogramming of fibroblasts into CAFs occurs through a two‐step process. Initially, cancer cells secrete exosomes that reprogram quiescent fibroblasts into activated CAFs. Subsequently, these cancer cells maintain the CAF phenotype by activating specific signal transduction pathways [220]. While this paradigm offers multiple therapeutic entry points, a major translational challenge lies in the inherent plasticity of CAFs—their ability to adapt to therapeutic pressure by switching between protumorigenic subtypes or developing compensatory signaling networks.

Existing strategies reverse the phenotype of CAFs by targeting key molecules or nanotechnology to reshape the immune microenvironment to enhance the response to immunotherapy. Converting CD146^−^CAFs into CD146^+^ CAFs represents a promising therapeutic strategy. Biffi et al. [20] demonstrated that JAK inhibitor‐mediated CAF reprogramming shifts iCAFs toward myCAFs, enhancing ECM deposition and suppressing tumor growth. Guo et al. [250] developed a Gemini‐like vitamin B3‐conjugated nanoparticle as a novel therapeutic approach for chemotherapy‐resistant cancers, enabling simultaneous CAF reprogramming and cancer cell elimination. Fei et al. [251] proposed a sequential nanodrug‐based therapy that reverses activated CAFs, normalizes tumor vasculature to alleviate hypoxia, improves tumor penetration of photosensitizers, and remodels the immunosuppressive TME, thereby enhancing photodynamic therapy efficacy in HCC. MyCAF‐secreted HA and iCAF‐derived hepatocyte growth factor drive the protumorigenic effects of CAFs, while myCAF‐expressed type I collagen counteracts these effects. This tumor‐restrictive role of collagen contrasts with the overall protumorigenic function of CAFs, providing mechanistic insight into their dual roles in cancer. Targeting protumorigenic CAF mediators while preserving type I collagen may offer an effective therapeutic strategy [63]. Wang et al. [252] revealed that SPP1^+^ myCAFs originate from iCAFs in hormone‐sensitive PCa, and this CAF subtype transition drives immune resistance. Targeting such stromal components could suppress castration‐resistant PCa progression. Current consensus suggests most CAF subtypes promote tumorigenesis, with only rare tumor‐suppressive subtypes like Meflin^+^ CAFs identified. Notably, Meflin^+^ CAFs differentiate into protumorigenic phenotypes during tumor progression. An ongoing clinical trial is evaluating Am80 (tamibarotene) for converting Meflin^−^ CAFs to Meflin^+^ CAFs, though further research on such strategies remains imperative [253].

In summary, restoring CAFs to a quiescent state to overcome immune resistance, particularly when combined with advanced nanomaterials, has demonstrated promising efficacy. These approaches highlight the therapeutic potential of targeting CAF plasticity and TME remodeling, yet challenges persist in deciphering dynamic CAF subtype interconversion and developing precise targeting strategies.

#### 6.2.3 Blocking the Crosstalk Between CAFs and TME

CAFs are recognized as pivotal components of the TME and play a crucial role in mediating immune resistance in tumors. By secreting various effector molecules, CAFs interact with tumor cells and immune cells, thereby shaping an immunosuppressive microenvironment. However, disrupting these complex interactions presents significant therapeutic challenges, as CAF‐derived signals often exhibit functional redundancy and context‐dependent effects. In breast cancer, four distinct CAF subtypes (CAF‐S1 to CAF‐S4) have been identified. The CAF‐S1 subset recruits CD4^+^ CD25^+^ T cells (n‐Tregs) via CXCL12 secretion, driving their differentiation into Foxp3^+^ Tregs, which in turn leads to the establishment of an immunosuppressive microenvironment [89]. The small molecule inhibitor plerixafor prevents CXCL12 from binding to its receptor, thus hindering signal transmission from CAFs to immune cells [254]. CAFs also facilitate the recruitment and differentiation of monocytes into protumor macrophage subpopulations, specifically M2‐TAMs, which impair effector T‐cell responses and induce immunosuppression within the TME [255]. In colon cancer, M2‐TAMs induced by CAFs exhibit high levels of PD‐1 on their surface [256]. A therapeutic strategy involves differentiating the M2 phenotype to the antitumor M1 phenotype, which can effectively slow tumor progression. Agents such as chlorogenic acid, CD40 agonist antibodies, histidine‐rich glycoprotein, and gemcitabine can polarize TAMs from the M2 phenotype to the M1 phenotype, thereby reversing antitumor immunity [257]. The therapeutic strategies mentioned above aim to modulate the interactions between CAFs and immune cells to restore or enhance antitumor immune responses within the TME, thereby improving the efficacy of cancer treatments. Specifically, they enhance the sensitivity and effectiveness of immunotherapy. Additionally, CAFs generate a plethora of inflammatory and cytokine factors through autophagy, which promotes the expression of immune checkpoints and impacts the function of immune cells, collectively contributing to the creation of an immunosuppressive microenvironment [258]. The application of autophagy inhibitors,  such as chloroquine or rapamycin, can regulate the autophagy process in CAFs, thereby overcoming drug resistance [259]. Current inhibitors lack the specificity required for clinical translation. More selective approaches targeting CAF‐specific autophagy components or utilizing stromal‐directed delivery systems may overcome these limitations.

Going forward, successful translation of CAF‐targeted strategies will necessitate addressing three key challenges: the dynamic redundancy of stromal signaling, the heterogeneity in CAF–immune interactions, and the need to preserve physiological immune function. Advances in real‐time monitoring of stromal activity, development of context‐dependent inhibitors, and optimization of sequential treatment regimens may help bridge the gap between promising preclinical results and meaningful clinical outcomes. By addressing these barriers, we can more effectively harness the potential of CAF‐TME modulation to enhance cancer immunotherapy.

### 6.3 Advancements in CAF Clinical Trials

Currently, clinical trials targeting CAFs mainly focus on the markers of CAFs and the related enriched pathways. The majority of clinical trials revolve around FAP and FGFR. We have summarized the clinical trial of CAFs in multiple cancers in Table 2. Nevertheless, the results of most current clinical trials involving CAFs have been unsatisfactory. This can be attributed to the inherent complexity and heterogeneity of CAFs [1]. Besides, the TME is an extremely complex system. Some clinical trials only target CAFs while neglecting the crosstalk between CAFs and other immunosuppressive cells, which might also account for the poor efficacy of some clinical trials. In the future, clinical trials should not merely concentrate on the markers and enriched pathways of CAFs; instead, a more comprehensive exploration of the role of CAFs in the TME is needed and the blocking of their crosstalk with immunosuppressive cells would be anticipated. Only by doing so can the therapeutic efficacy of clinical trials be more significantly enhanced.

**TABLE 2:** Clinical trials targeting CAFs.

Target	Tumor type	Phase	Drug	Combination agent	Clinical efficacy	NCT number	Status	References
FAP	Malignant tumor	Not applicable	68Ga‐FAPI	−	−	NCT05034146	Recruiting	−
		Not applicable	FAPI‐74	−	−	NCT05442151	Recruiting	−
		Not applicable	68Ga‐FAPI	−	−	NCT04554719	Unknown status	−
		Not applicable	68Ga‐FAPI	−	−	NCT05263700	Unknown status	−
		Phase 1	FAP‐2286	−	−	NCT04621435	Recruiting	[1]
		Phase 1	68Ga‐FAPi‐46	−	−	NCT04459273	Recruiting	[1]
		Phase 1	OMTX705	Pembrolizumab	−	NCT05547321	Recruiting	−
		Phase 1/2	68Ga‐FAP‐2286	177Lu‐FAP‐2286	−	NCT04939610	Recruiting	−
		Phase 2	RO6874281	Atezolizumab Gemcitabine Vinorelbine	−	NCT03386721	Terminated	−
		Not applicable	18F‐FAPI	18F‐FDG	−	NCT05485792	Unknown status	−
	PDAC^a^	Not applicable	68Ga‐FAPI‐46	−	−	NCT06911021	Not recruiting	−
		Phase 2	68Ga‐FAPI‐46	−	−	NCT05518903	Recruiting	−
		Not applicable	68Ga‐FAPI	−	−	NCT05275985	Unknown status	−
	PDAC^a^/OGA^b^	Phase 2/3	AlF‐FAPI‐74	−	−	NCT06782412	Recruiting	−
	GC^c^	Phase 2	FAPI‐74	−	−	NCT05641896	Recruiting	[1]
		Not applicable	18F‐FAPI	18F‐FDG	−	NCT06327386	Recruiting	−
	LUAD^d^	Not applicable	68Ga‐FAPI	−	−	NCT06107608	Recruiting	−
	HCC^g^	Phase 1	68Ga‐FAPI‐46	−	−	NCT05687747	Recruiting	−
	OC^h^	Not applicable	68Ga‐FAPI	−	−	NCT05856409	Recruiting	−
		Phase 2	68Ga‐FAPI‐04	18F‐FDG	−	NCT04504110	Unknown status	[1]
	BC^i^	Not applicable	Al18F‐NOTA‐FAPI‐04	−	−	NCT05574920	Not recruiting	−
	ICC^j^	Phase 2/3	[18F]‐FAPI	−	−	NCT06355427	Recruiting	−
	CRC^k^	Not applicable	FAPI	−	−	NCT05209750	Recruiting	−
		Not applicable	[18F]‐ALF‐FAPI‐74	−	−	NCT06191120	Not recruiting	−
	PC^l^	Not applicable	FAPI	−	−	NCT06634173	Not recruiting	−
	OSCC^m^	Not applicable	68Ga‐DOTA‐FAPI	18F‐FDG	−	NCT05030597	Unknown status	−
	HNSC^o^	Phase 2	[68Ga]‐FAPI‐46	−	−	NCT06794372	Not recruiting	−
FGFR^e^	Malignant tumor	Phase 1	Rogaratinib	−	−	NCT01976741	Completed	−
		Phase 2	Pemigatinib	−	−	NCT03822117	Terminated	−
		Phase 1	JNJ‐42756493	−	JNJ‐42756493 administered at 10 mg on a 7‐days‐on/7‐days‐off schedule achieved exposures at which clinical responses were observed, demonstrated pharmacodynamic biomarker activity, and had a manageable safety profile.	NCT01703481	Completed	−
		Phase 1/2	Futibatinib	−	Futibatinib treatment resulted in manageable safety, pharmacodynamic activity, and preliminary responses in patients with advanced solid tumors. The results of this phase I dose‐escalation trial support 20 mg q.d. futibatinib as the RP2D.	NCT02052778	Completed	−
		Phase 1/2	Derazantinib	−	ARQ 087 had manageable toxicity at the RP2D of 300 mg QD, showed pharmacodynamics effects, and achieved objective responses, notably in patients with FGFR2 genetic alterations.	NCT01752920	Completed	−
	PDAC^a^	Not applicable	Sorafenib	Gefitinib	−	NCT06592989	Recruiting	−
		Phase 1/2	BGJ398	Fluorouracil Irinotecan Oxaliplatin	−	NCT02575508	Withdrawn	−
	LUAD^d^	Phase 1	GSK3052230	Paclitaxel Carboplatin Docetaxel Pemetrexed Cisplatin	−	NCT01868022	Completed	−
		Phase 2	Pemigatinib	−	−	NCT05253807	Completed	−
	BLCA^f^	Phase 2	Pemigatinib	−	−	NCT03914794	Recruiting	−
		Phase 1/2	Erdafitinib	−	−	NCT05316155	Recruiting	−
		Not applicable	Balversa	−	−	NCT05052372	Terminated	−
		Phase 3	Infigratinib	Placebo	−	NCT04197986	Terminated	−
	HCC^g^	Phase 1	INCB062079	−	−	NCT03144661	Terminated	−
	OC^h^	Phase 1/2	Pamiparib	Surufatinib	−	NCT05494580	Recruiting	−
	ICC^j^	Phase 2	E7090	−	−	NCT04238715	Not recruiting	−
	UC^n^	Phase 2	Pemigatinib	−	−	NCT02872714	Completed	−
TGF‐β	PDAC^a^	Phase 1/2	Tamibarotene	Gemcitabine Nab‐Paclitaxel	−	NCT05064618	Recruiting	−
	BC^i^	Phase 1	Galunisertib	Paclitaxel	−	NCT02672475	Not recruiting	[1]
		Phase 2	Fresolimumab	−	Abscopal response rate 100% for group: fresolimumab 1 mg/kg and group; fresolimumab 10 mg/kg	NCT01401062	Completed	[1]
	CRC^k^	Phase 1/2	LY3200882	Capecitabine	−	NCT04031872	Unknown status	[1]
PD‐L1	Malignant tumor	Phase 1	SAR439459	Cemiplimab	−	NCT03192345	Terminated	[1]
		Phase 1	SAR439459	Cemiplimab	−	NCT04729725	Terminated	[1]
		Phase 1/2	LY2157299	Nivolumab	In phase 2, researchers provide the date on ORR, ORR of galunisertib + nivolumab (NSCLC) group and galunisertib + nivolumab (HCC) group was 24 and 0%	NCT02423343	Completed	[1]
	BC^i^	Phase 1	M7824	−	−	NCT03620201	Not recruiting	[1]
	UC^n^	Phase 2	TEW‐7197	Durvalumab	−	NCT04064190	Not recruiting	[1]
	OPL^p^	Phase 2	Avelumab	−	−	NCT04504552	Unknown status	−
Type I collagen	HNSC^o^	Phase 2	Pirfenidone	Placebo	−	NCT06142318	Recruiting	−
CXCR4	Malignant tumor	Phase 1	MSX‐122	−	−	NCT00591682	Suspended	[1]
	PDAC^a^	Phase 2	AMD3100	Cemiplimab	−	NCT04177810	Completed	[1]
		Phase 2	BL‐8040	Pembrolizumab	In cohort 1, the DCR was 34.5% in the evaluable population (modified intention to treat, mITT; *N* = 29), including nine patients (31%) with stable disease and one patient (3.4%) with partial response. In cohort 2, 22 patients received BL‐8040 and pembrolizumab with chemotherapy, with an ORR, DCR, and median duration of response of 32%, 77%, and 7.8 months, respectively.	NCT02826486	Completed	[1]
		Phase 2	Cemiplimab	Gemcitabine Nab‐Paclitaxel	−	NCT04543071	Recruiting	[1]
	PDAC^a^/LUAD^d^	Phase 1/2	BMS‐936564	Nivolumab	−	NCT02472977	Terminated	[1]
	BC^i^	Phase 1	POL6326	Eribulin	Objective responses (all partial responses) were observed in 16 (30%; 95% CI 18–44) of 54 patients who were evaluable for antitumor activity	NCT01837095	Completed	[1]
LRRC15	Malignant tumor	Phase 1	ABBV‐085	−	In the “all sarcomas at all doses” population the ORR was 10.8%. In the patients with osteosarcoma or UPS treated at the 3.6 mg/kg dose the ORR was 20%. Among patients with UPS treated at 3.6 mg/kg, four patients had PR with tumor shrinkage of >30%.	NCT02565758	Completed	[1]
PI3K/AKT	Malignant tumor	Phase 1	CUDC‐907	−	−	NCT02307240	Completed	[1]
STAT3	Malignant tumor	Phase 1/2	BBI503/BBI608	Paclitaxel	Researchers provided data on ORR and DCR. ORR among five arms was 0, 28.4, 0, 9.2, and 20.7%, respectively; DCR among five arms was 100, 53.1, 0, 56.1, and 51.7%, respectively.	NCT01325441	Completed	[1]
	PDAC^a^	Phase 3	BBI608	Gemcitabine Nab‐Paclitaxel	DCR among napabucasin‐treated and control‐treated patients was 74.5 and 76.0%, respectively, and ORR was 43.2 and 42.9%, respectively.	NCT02993731	Completed	[1]
	LUAD^d^	Phase 3	BBI608	Paclitaxel	−	NCT02826161	Terminated	[1]
	HCC^g^	Phase 1/2	BBI503/BBI608	Sorafenib	Researchers provided data on ORR and DCR in phase 2 study. ORR based on RECIST 1.1 criteria among three arms was 3.6% (95% CI 0.1–18.3%), 0 (95% CI 0–30.8%), 9.7% (95% CI 2–25.8%), respectively. DCR based on RECIST 1.1 criteria among three arms was 35.7% (95% CI 18.6–55.9%), 70% (95% CI 34.8–93.3%), 48.4% (95% CI 30.2–66.9%), respectively.	NCT02279719	Completed	[1]

*Note*: a: pancreatic ductal adenocarcinoma, b: esophagogastric adenocarcinoma, c: gastrointestinal cancer, d: lung adenocarcinoma, e: fibroblast growth factor receptor, f: bladder urothelial carcinoma, g: hepatocellular carcinoma, h: ovarian cancer, i: breast cancer, j: intrahepatic cholangiocarcinoma, k: colorectal carcinoma, l: prostate cancer, m: oral squamous cell carcinoma, n: urothelial cancer, o: head and neck squamous cell carcinoma, p: oral premalignant lesions.

*Data sources*: https://clinicaltrials.gov/.

## 7 Conclusion and Prospects

The comprehensive analysis presented in this review underscores the pivotal role of CAFs as master regulators of tumor progression, immune evasion, and therapy resistance. Through their remarkable heterogeneity and dynamic interactions within the TME, CAFs have emerged as central players in shaping both tumor‐promoting and immunosuppressive niches. Our systematic evaluation of CAF origins, activation mechanisms, and functional classification provides a framework for understanding their diverse roles across cancer types. The identification of eight distinct CAF subtypes, characterized by unique molecular signatures and spatial distributions, represents a significant advance in stromal biology and offers new opportunities for targeted therapeutic interventions.

Despite significant progress, several critical challenges remain unresolved in CAF research. First, the plasticity and context‐dependent functions of CAF subtypes necessitate more precise tools for their identification and tracking in vivo. While single‐cell technologies have revolutionized CAF characterization, the field still lacks consensus markers that can reliably distinguish tumor‐promoting from tumor‐restraining CAF populations across different malignancies. Second, the bidirectional crosstalk between CAFs and other TME components creates complex feedback loops that are not fully understood. For instance, how specific CAF subtypes dynamically adapt their secretory profiles in response to therapy‐induced TME remodeling requires further investigation. Third, the metabolic interplay between CAFs and tumor cells presents both challenges and opportunities—while we have identified key metabolic pathways involved in CAF‐mediated immune suppression, translating these findings into clinically viable strategies remains difficult due to systemic toxicity concerns.

The integration of cutting‐edge technologies will be instrumental in addressing these challenges. Spatial multiomics platforms that combine transcriptomic, proteomic, and metabolomic data with cellular resolution are poised to uncover new dimensions of CAF heterogeneity. Advanced 3D models incorporating patient‐derived CAFs and immune cells will better recapitulate the human TME for mechanistic studies and drug screening. Artificial intelligence approaches, particularly deep learning algorithms trained on large‐scale CAF datasets, could predict subtype‐specific vulnerabilities and optimize combination therapies. Importantly, the development of humanized mouse models with engineered CAF subpopulations will provide much‐needed preclinical systems for validating therapeutic targets while accounting for human‐specific stromal biology.

Translating our growing understanding of CAF biology into clinical applications represents both promise and complexity. Current strategies focusing on CAF depletion have shown limited success, underscoring the need for more nuanced approaches. Future directions should emphasize selective targeting of pathogenic CAF subsets while preserving tumor‐suppressive populations, disruption of specific CAF–immune cell axes driving resistance (e.g., CXCL12–CXCR4 or TGF‐β signaling), and metabolic reprogramming of CAFs to reverse immunosuppression. The development of dual‐targeting agents that simultaneously modulate CAFs and enhance immune cell function—such as bifunctional TGF‐β/PD‐L1 inhibitors—appears particularly promising. Additionally, nanoparticle‐based delivery systems that preferentially accumulate in CAF‐rich stroma could improve therapeutic precision while reducing off‐target effects. Clinical trials must incorporate robust CAF biomarkers and spatial profiling to stratify patients and monitor stromal reprogramming in real time.

Looking ahead, five priority areas will shape CAF research: establishing universal subtyping criteria across malignancies, elucidating epigenetic control of CAF plasticity, developing dynamic models of CAF evolution during therapy, exploring nervous system–CAF crosstalk, and harnessing CAF signatures as predictive biomarkers. The field must progress from observational studies to establishing causal relationships between CAF programs and clinical outcomes. Through interdisciplinary collaboration, we can transform CAF biology from a complex challenge into a therapeutic opportunity that complements existing cancer treatments. This review not only consolidates current understanding but also charts a roadmap for future research to overcome stromal‐mediated therapy resistance and unlock the full potential of TME‐targeted therapies.

## Author Contributions

Chang Fan, Wenlong Zhu, and Yuan Chen searched the literature and drafted the manuscript. Jin Ding and Wanwan Zhu made critical revisions of the manuscript. All the authors acknowledged the contributors and have read and approved the final manuscript.

## Conflicts of Interest

The authors declare no conflicts of interest.

## Ethics Statement

No ethical approval was required for this study.

## Data Availability

No data were used in the article.
